# BAL Proteomic Signature of Lung Adenocarcinoma in IPF Patients and Its Transposition in Serum Samples for Less Invasive Diagnostic Procedures

**DOI:** 10.3390/ijms24020925

**Published:** 2023-01-04

**Authors:** Lorenza Vantaggiato, Enxhi Shaba, Paolo Cameli, Laura Bergantini, Miriana d’Alessandro, Alfonso Carleo, Giusy Montuori, Luca Bini, Elena Bargagli, Claudia Landi

**Affiliations:** 1 Functional Proteomic Section, Department of Life Sciences, University of Siena, 53100 Siena, Italy; 2 UOC Respiratory Diseases and Lung Transplantation, Department Internal and Specialist Medicine, University of Siena, 53100 Siena, Italy; 3 Department of Pneumology, Medical School Hannover (MHH), 30539 Hannover, Germany

**Keywords:** BALF, IPF, lung cancer, proteomics, biomarkers, profilin 1, NAMPT, Apolipoprotein A1, Apolipoprotein E, Calgranulin B

## Abstract

Idiopathic pulmonary fibrosis (IPF) is a form of chronic and irreversible fibrosing interstitial pneumonia of unknown etiology. Although antifibrotic treatments have shown a reduction of lung function decline and a slow disease progression, IPF is characterize by a very high mortality. Emerging evidence suggests that IPF increases the risk of lung carcinogenesis. Both diseases show similarities in terms of risk factors, such as history of smoking, concomitant emphysema, and viral infections, besides sharing similar pathogenic pathways. Lung cancer (LC) diagnosis is often difficult in IPF patients because of the diffuse lung injuries and abnormalities due to the underlying fibrosis. This is reflected in the lack of optimal therapeutic strategies for patients with both diseases. For this purpose, we performed a proteomic study on bronchoalveolar lavage fluid (BALF) samples from IPF, LC associated with IPF (LC-IPF) patients, and healthy controls (CTRL). Molecular pathways involved in inflammation, immune response, lipid metabolism, and cell adhesion were found for the dysregulated proteins in LC-IPF, such as TTHY, APOA1, S10A9, RET4, GDIR1, and PROF1. The correlation test revealed a relationship between inflammation- and lipid metabolism-related proteins. PROF1 and S10A9, related to inflammation, were up-regulated in LC-IPF BAL and serum, while APOA1 and APOE linked to lipid metabolism, were highly abundant in IPF BAL and low abundant in IPF serum. Given the properties of cytokine/adipokine of the nicotinamide phosphoribosyltransferase, we also evaluated its serum abundance, highlighting its down-regulation in LC-IPF. Our retrospective analyses of BAL samples extrapolated some potential biomarkers of LC-IPF useful to improve the management of these contemporary pathologies. Their differential abundance in serum samples permits the measurement of these potential biomarkers with a less invasive procedure.

## 1. Introduction

Idiopathic pulmonary fibrosis (IPF) is a specific form of chronic, progressive, and fibrosing interstitial pneumonia of unknown cause, which mostly occurs in elderly males who have a smoking habit [1,2,3]. It is characterized by progressive worsening of dyspnoea and lung function, associated with a poor prognosis [4]. 

Antifibrotic therapies decelerate the disease progression and, consequently, increase the survival of IPF patients. However, increased survival due to delayed disease progression leads to a higher prevalence of comorbidities and complications [5]. Indeed, many patients with IPF also have other comorbid conditions that include emphysema, pulmonary hypertension, sleep apnoea, coronary artery disease, and lung cancer (LC) [6]. In particular, recent meta-analyses reported close associations between the development of IPF and LC [7,8,9,10] and IPF itself increases the risk of LC development from 7% to 20% [11]. IPF patients that are diagnosed with LC have a reduced mean survival time (1.6–1.7 years), compared to IPF patients without LC [7,12,13]. In particular, non-small cell lung cancer (NSCLC) is the predominant type of LC in IPF patients and adenocarcinoma (ADC) is the most common subtype of histological NSCLC in the general population [14].

Both IPF and LC often affect the periphery of lower lung lobes and share common risk factors such as smoking, environmental or occupational exposure, viral infections, and chronic tissue injury. Moreover, IPF and LC studies also report common pathogenic mechanisms such as epigenetic and genetic alterations, abnormal expression of microRNAs, and cell and molecular aberrations [6,11,15]. In particular, specific signalling transduction pathways such as myofibroblast/mesenchymal transition, myofibroblast activation and uncontrolled proliferation, endoplasmic reticulum stress, alterations of growth factors expression, and oxidative stress [11,16,17] are activated in both the pathologies. Pathogenetic similarities between IPF and LC represent a fundamental point for common therapeutic approaches, such as the use of nintedanib [16,18], reported as both an anti-fibrotic and anti-cancer agent [19]. In previous work, we showed as the active substance of nintedanib acts on some molecular pathways shared between IPF and LC [16]. Unfortunately, this therapy, as well as other antifibrotic and anti-oncogenic drugs such as pirferidone, have not been tested prospectively in patients with coexisting IPF and lung cancer [18]. Therefore, questions regarding the proper and ideal management of patients who suffer from both IPF and LC are also raised. Biomarker research to highlight diagnostic, prognostic, and therapeutic markers of diseases could be useful to improve the management of these contemporary severe pathologies. These kinds of data on patients with IPF and LC are still scarce. Proteomics applied to bronchoalveolar lavage fluid (BALF) was already used to discover biomarkers of different interstitial lung diseases (ILDs) [2,3,20,21,22,23] as well as to stratify different ILD phenotypes [24,25]. For this purpose, we firstly aimed to extrapolate potential protein biomarkers of LC onset by proteomic approach. BAL samples from IPF, LC associated with IPF (LC-IPF) patients, and healthy controls (CTRLs) were resolved by two dimensional electrophoresis (2DE). Once the 2DE-gels were obtained, we performed an image comparison in order to detect differential abundant spots between CTRLs and IPF, CTRLs and LC-IPF, and, finally, IPF and LC-IPF. Then, spots were identified by mass spectrometry. Identified proteins were submitted to enrichment analysis by bioinformatics approach This step provides molecular pathways where these proteins are involved. By enrichment results, relevant proteins for IPF and especially for LC onset in IPF patients were also evaluated by Western blot in other BALF samples. Moreover, we extended the evaluation in serum samples of the same patients in order to develop diagnostic instruments requiring a less invasive procedure to assess the pathological status, more suitable for such compromised patients. Finally, we performed a correlation analysis between differential abundant proteins found by proteomics and clinical data. 

## 2. Results

### 2.1. Population

The main demographic, clinical, respiratory functional and BAL data of IPF, LC-IPF, and healthy controls are reported in Table 1. Healthy controls were significantly younger than IPF patients, but not in respect to LC-IPF: no differences were reported among all subgroups in terms of gender and smoking status. Respiratory functional assessment, including DLCO, and BAL features were comparable between IPF and LC-IPF patients.

Histological and/or cytological evaluation of sampling obtained through CT-guided needle aspiration, transbronchial biopsy, and drainage of malignant pleural effusion showed a pattern of lung adenocarcinoma in all LC-IPF cases.

### 2.2. Proteomic and Multivariate Analyses Revealed a Differential BALF Protein Pattern between IPF and LC-IPF

Proteomic analysis was performed matching all 2DE-gels obtained from BALF samples of the three analysed conditions (IPF, LC-IPF, and CTRL). Spot data by proteomics permitted us to perform an unsupervised multivariate analysis by heatmap and principal component analysis (PCA), as shown in Figure S1A and S1B, respectively. Protein abundance of all matched spots in all gels immediately shows an opposite behavior between all IPF and CTRLs without evident distinction between IPF and LC-IPF.

**Figure 1:**
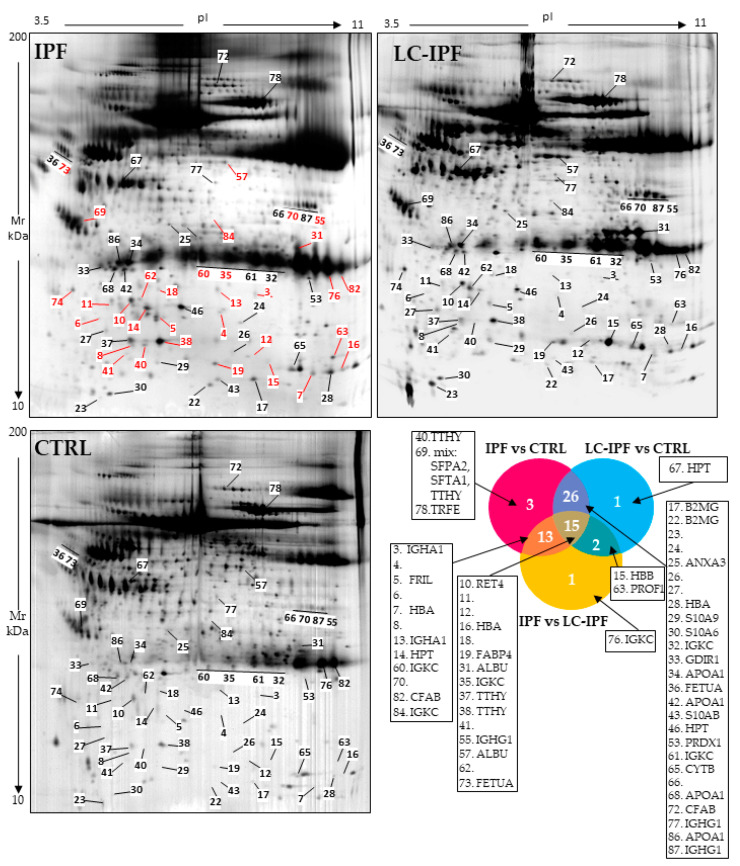
IPF, LC-IPF, and CTRL BALF reference gel maps reporting the statistically relevant spots with a fold change ratio ≥1.8. Red spots represent differences between IPF and LC-IPF. Moreover, Venn diagram shows 13 overlapping proteins between the IPF vs. LC-IPF and IPF vs. CTRL comparisons; 2 overlapping proteins between the LC-IPF vs. CTRL and IPF vs. LC-IPF comparisons; 26 overlapping proteins between the IPF vs. CTRL and LC-IPF vs. CTRL comparisons; and 15 overlapping proteins among the three comparisons. Only one protein was exclusively associated with IPF vs. LC-IPF and IPF vs. CTRL comparisons. Relative proteins are reported in the side panels.

In order to extrapolate differential abundant spots with high significance among the conditions, we performed Kruskal–Wallis analysis taking into consideration only spots with a *p*-value ≤ 0.05 and percentage of relative volume (%V) mean ratio ≥1.8. Sixty-one spots satisfying these parameters are shown on the reference gel images in Figure 1, where 31 differential spots between IPF and LC-IPF are reported in red. Figure 1 also reports the Venn diagram, showing the differential protein spots in common among the comparisons: 13 in IPF vs. LC-IPF/IPF vs. CTRL, 26 in IPF vs. CTRL/LC-IPF vs. CTRL, 15 among the three comparisons and 2 in IPF vs. LC-IPF/LC-IPF vs. CTRL. Identified proteins referred to Venn’s groups are reported in the side panels. Table S1 reports data referred to statistical analysis with spot number (Figure 1), UniProt Protein name, accession number, Kruskal–Wallis results, means, and fold ratio results, while Table 2 further reports the Mascot Search Results after protein identification by peptide mass fingerprint (PMF). 

**Figure 2:**
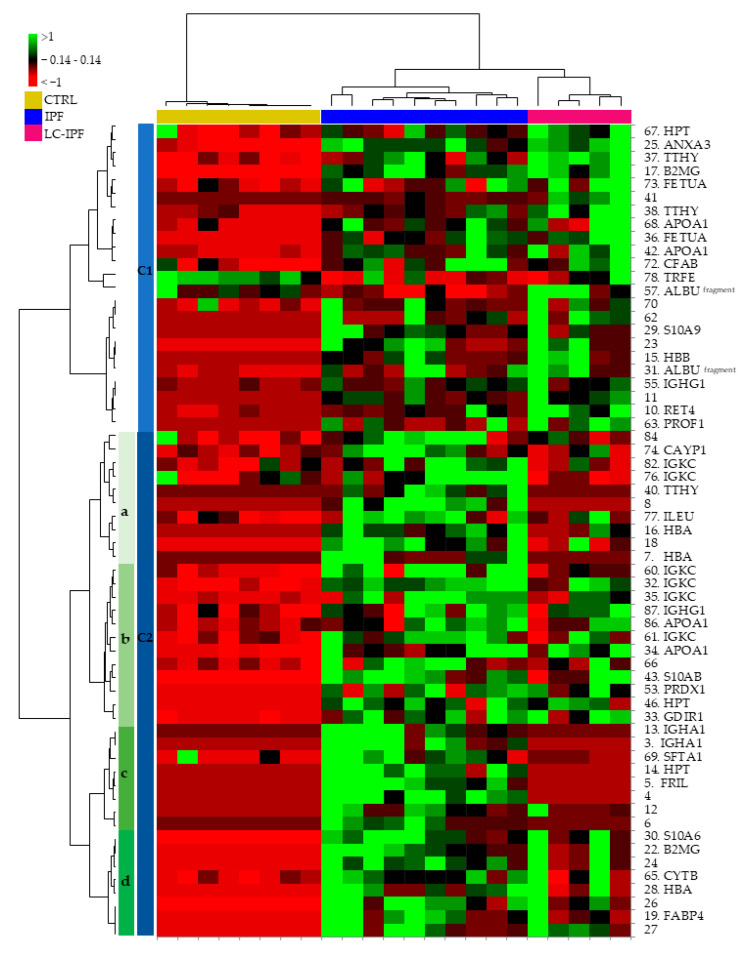
Supervised heatmap analysis by XLSTAT performed using the statistically valid DASs, detected by proteomic analysis of BALF samples.

**Figure 3:**
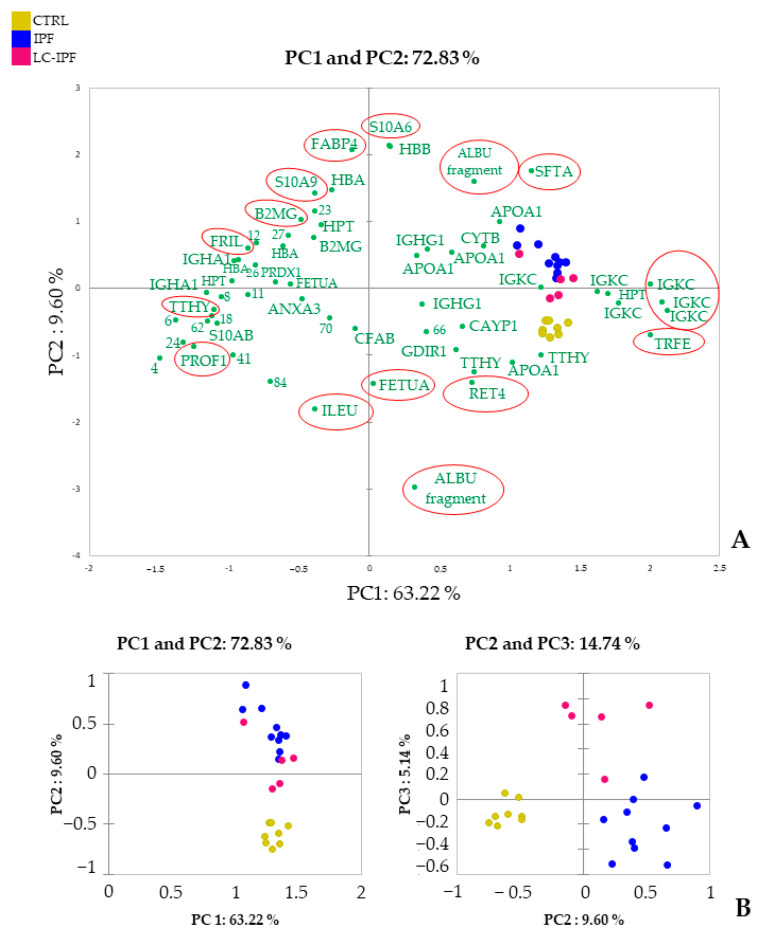
Supervised PCA analysis performed using the DASs detected by proteomic analysis of BALF samples. (**A**). The image reports the contributions of each significant variant in the first two principal components (PC1 and PC2). Red circles mark the most influential spots on the samples distributions. (**B**). Plots highlight the spatial distribution of the analyzed samples along the PC1 and PC2, and PC1 and PC3.

Based on the 61 differentially abundant spots (DASs), we elaborated a further supervised multivariate analysis by heatmap and PCA, as reported in Figure 2 and Figure 3, respectively. In this case, both the heatmap and PCA show differences in protein trend, not only between all IPF and CTRLs, but also between IPF and LC-IPF. Heatmap analysis in Figure 2 clusters the DASs into two principal clusters (C1 and C2): C1 highlights the highly abundant proteins in LC-IPF, such as HTP, ANXA3, TTHY, B2MG, FETUA, APOA1, CFAB, ALBU (fragment), S10A9, HBB, IGHG1, RET4, and PROF1, with the exception of TRFE which is the only protein species resulting as down-regulated. In particular, C2a and C2c show the highly abundant protein species in IPF, composed of CAYP1, IGKC, TTHY, HBA, ILEU, IGHA1, SFTA, and FRIL, while C2b and C2d group gather the protein species more abundant in both IPF and LC-IPF compared to CTRLs, such as IGKC, APOA1, S10AB, PRDX1, HPT, GDIR1, S10A6, B2MG, CYTB, HBA, and FABP4. PCA shows a variance of 72.83% (PC1 63,22% and PC2 9.60%) (Figure 3), also showing the contributions of each significant variant (proteins) in the first two PCs. Specifically, PROF1, TTHY, FRIL, B2MG, S10A9, FABP4, S10A6, ALBU fragments, SFTA, TRFE, RET4, FEUA, and ILEU, circled in red, show a high contribution to samples stratification in PCA.

### 2.3. Enrichment Analysis Discovered Dynamics of Inflammation, Lipid Metabolism, and Cell Adhesion Biomarkers in IPF and LC-IPF

Enrichment analysis was performed by MetaCore software uploading the identified proteins in BALF samples by proteomic analysis, divided into different lists based on their abundance trend.

**Figure 4:**
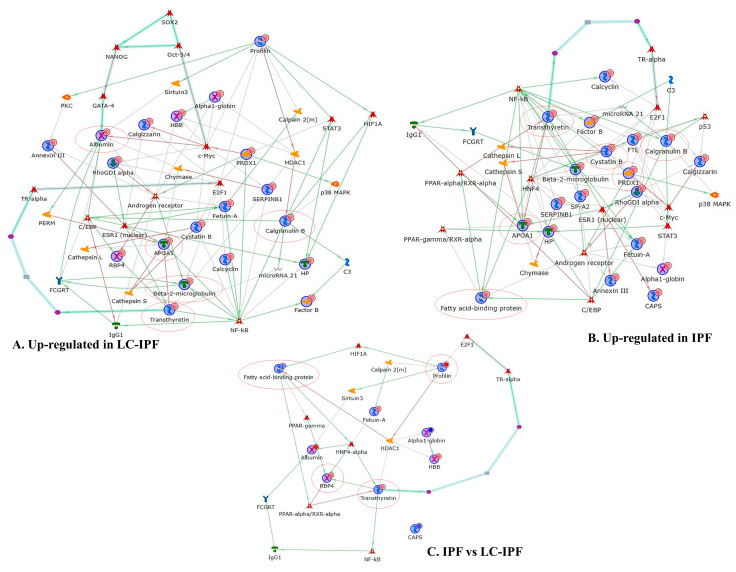
(**A**) Protein network of the up-regulated proteins in LC-IPF with respect to CTRL, and IPF BALF samples. Albumin, Calgranulin B, APOA1, Transthyretin, and Beta-2-microglobulin are the respective central hubs. (**B**) Protein network of the up-regulated proteins in IPF with respect to CTRL and LC-IPF. APOA1, Calgranulin B, Transthyretin, Cystatin B, and PRDX1 are the central hubs. (**C**) Differential proteins between IPF and LC-IPF. Transthyretin, Profilin I, RBP4, A-FABP, and IGHG1 are central hubs (marked by a red circle).

In order to understand the interaction between the highly abundant proteins in IPF or in LC-IPF, we performed two independent protein network analyses. Figure 4A reports the protein network built by proteins with progressively increasing abundance from CTRL to IPF to LC-IPF. In this network, ALBU, S10A9, PROF1, TTHY, APOA1, and B2MG result in central functional hubs, i.e., proteins with the highest number of interactions with the other proteins in the net, and, for this reason, probable pivotal modulators of up-/down-stream molecular pathways. Transcriptional factors suggested by MetaCore, which are related to the central hubs, are GATA-4, c-Myc, C/EBP, Androgen receptor, NF-kB, ESR1, E2F1, TR-alpha, STAT3, and HIF-1A. The protein network also evidences two canonical paths (turquoise bold lines): one starting from c-myc to albumin and the other starting from transthyretin to E2F1 transcriptional factor. On the other hand, Figure 4B reports the protein network performed with the higher abundant proteins in IPF with respect to CTRL and LC-IPF. The protein network shows APOA1, S10A9, TTHY, CYTB, and PRDX1 as central hubs. These central hubs are connected with transcriptional factors such as NF-kB, Androgen receptor, c-Myc, ESR1, E2F1, TR-alpha, STAT3, and C/EBP in common with the network in Figure 4A, and with HNF4, p53, PPAR-alpha/RXR-alpha, and PPAR-gamma/RXR-alpha, specific to IPF. In this case, the canonical path starting from transthyretin to E2F1 is reported. Moreover, we performed the protein network with the differential proteins found between IPF and LC-IPF in order to highlight specific biomarkers for LC-IPF with respect to IPF (Figure 4C). The bubbles color, which marks the proteins, changes from blue to red, indicating less or higher protein abundance in LC-IPF compared to IPF, respectively. FABP4, PROF1, RET4 and TTHY are central hubs linked to transcriptional factors such as PPAR-gamma and alpha, HIF1A, HNF4-alpha, NF-kB, TR-alpha, and E2F1. In this case a canonical path starting from transthyretin to E2F1 is evidenced. 

**Figure 5:**
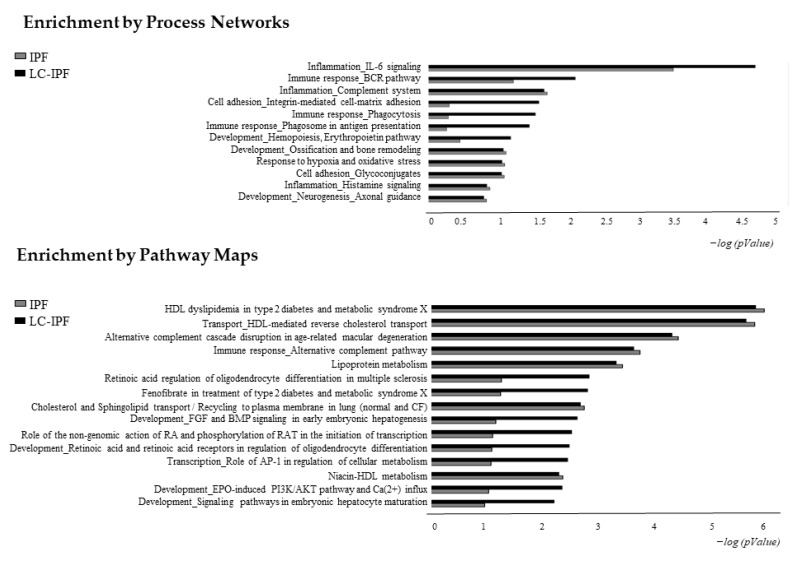
Histograms reporting the Process Networks and Pathway Maps comparisons of up-regulated proteins in IPF and in LC-IPF. Black histograms represent the −log(*p*-value) related to up-regulated proteins in LC-IPF BALF; grey histograms show the −log (*p*-value) related to up-regulated proteins in IPF BALF.

Furthermore, in order to distinguish experimentally validated biological functions and canonical maps associated with the highly abundant proteins in IPF or LC-IPF, we performed the Process Network and Pathway Maps comparisons by MetaCore software (Figure 5). The most statistically significant process networks of the up-regulated proteins in LC-IPF are inflammation related to IL6 signalling, immune response related to BCR pathway, phagocytosis and phagosome antigen presentation, cell adhesion by integrin mediated cell-matrix adhesion, and development by erythropoietin pathway. The process networks mostly associated with the highly abundant proteins in IPF are inflammation related to complement system and histamine signalling, response to hypoxia and oxidative stress, cell adhesion by glycoconiugates, development by neurogenesis, and ossification and bone remodelling (Figure 5 and Table S2, Supplemental Materials). Pathway Maps analysis reveals HDL dyslipidemia, HDL-mediated reverse cholesterol transport, immune response by alternative complement pathway, lipoprotein metabolism, cholesterol and sphingolipid transport with a similar significance for both LC-IPF and IPF. On the other hand, retinoic acid regulation and its role in the initiation of transcription and development, and the role of AP-1 in regulation of cellular metabolism are mostly related to LC-IPF (Figure 5). Proteins related to these pathways are reported in Table S3.

### 2.4. Western Blot Analyses in BALF and Serum Samples of APOAI, APOE, PROF1 and S10A9, Pivotal in IPF and/or LC-IPF 

**Figure 6:**
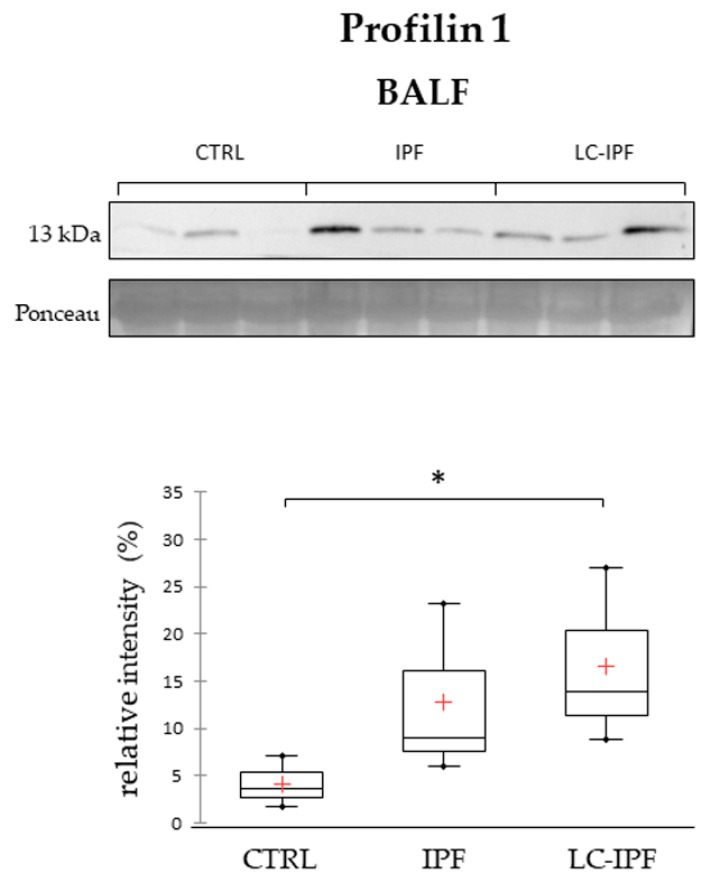
Western blot analysis of profilin 1 in BALF samples. Statistical analysis was performed by Kruskal–Wallis test and Dunn correction and reported in the Box plots (^∗^ *p*-value < 0.05). Ponceau red is reported to assure the equal loaded amount of sample and for intensity normalization. BALF samples used for WB were three for CTRL, three IPF, and three for LC-IPF. Samples used were three for CTRL, three IPF, and three for LC-IPF.

Given the role of centrals hub of PROF1, highly abundant in LC-IPF, in the protein net in Figure 4C, we decided to investigate its trend of abundance by Western blot in alternative BALF and serum samples from patients with IPF, LC-IPF, and healthy donors, in order to understand its potentiality as LC-IPF biomarker. As shown in Figure 6, PROF1 tested in BALF samples shows a slight increase in LC-IPF condition with respect to IPF and even more extensive compared to CTRL. Unfortunately, WB analysis in serum samples does not reveal any signal for PROF1. 

**Figure 7:**
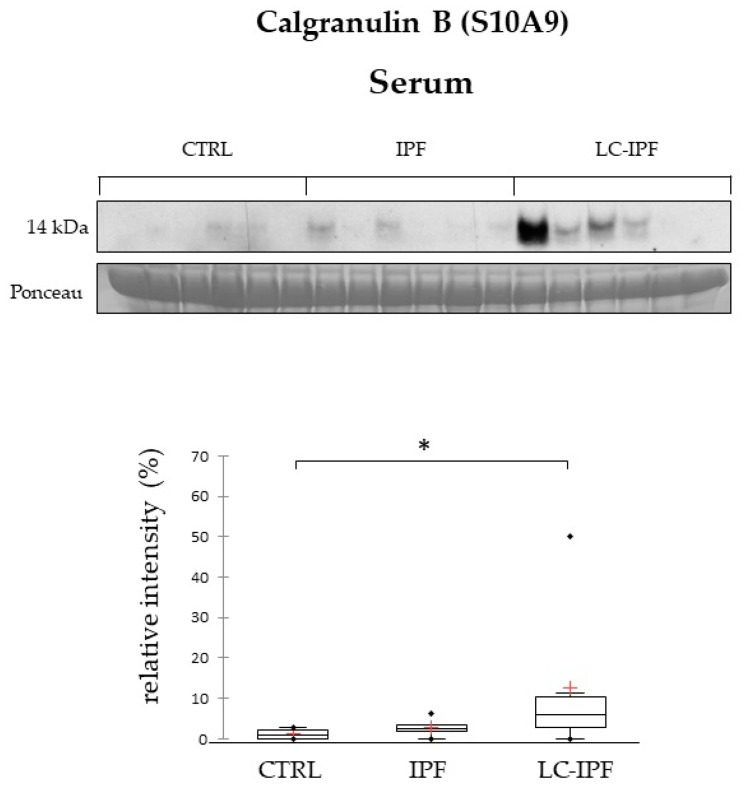
Western blot analysis of calgranulin B in serum samples. Statistical analysis was performed by Kruskal–Wallis test and Dunn correction and reported in the Box plots (^∗^ *p*-value < 0.05). Ponceau red is reported to assure the equal loaded amount of sample and for intensity normalization. Serum samples used for WB were six for CTRL, six for IPF and six for LC-IPF.

Moreover, S10A9 was found to be a central hub in the protein nets (Figure 4A,B), and also to be involved in inflammatory pathways. By proteomic analysis, S10A9 is reported as highly abundant in IPF BALF samples and even more in LC-IPF. Indeed, its evaluation at the serum level shows the same trend of abundance, confirming that S10A9 could be measured with a less invasive procedure to distinguish IPF from LC-IPF patients (Figure 7). 

**Figure 8:**
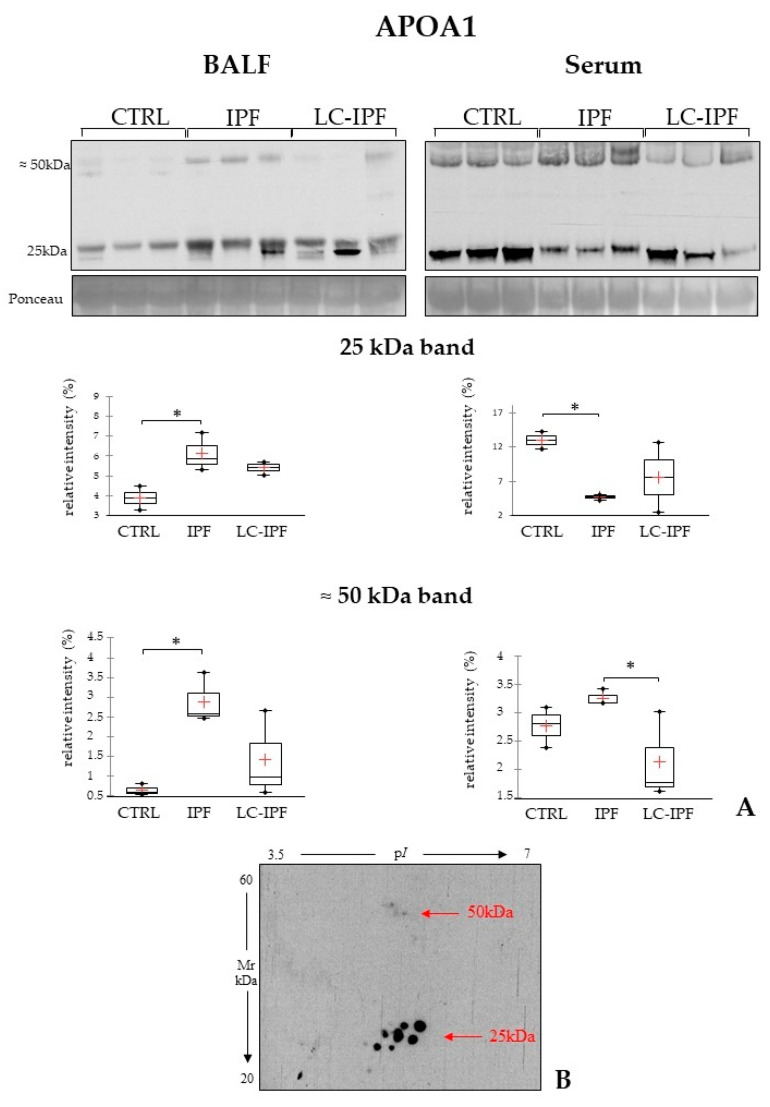
(**A**). Western blot analysis of APOA1 in BALF and serum samples. Statistical analysis was performed by Kruskal–Wallis test and Dunn correction and reported in the Box plots (^∗^ *p*-value < 0.05). Ponceau red is reported to assure the equal loaded amount of sample and for the intensity normalization. BALF and serum samples used for WB were three for CTRL, three for IPF, and three for LC-IPF. (**B**). In the bottom, the two-dimensional Western blot of APOA1 in an IPF BAL sample is reported. Classical protein species of APOA1 are highlighted at 25 kDa and other protein species at higher molecular weight (50 kDa) are also evidenced in IPF.

HDL dyslipidemia and lipoprotein metabolism are common dysregulated pathways suggested by the up-regulated proteins in IPF and in LC-IPF. APOA1 plays a central role in these pathways, and interestingly we identified an increase in abundance in IPF and LC-IPF with respect to CTRLs of different proteoforms of APOA1. To this purpose, we tested APOA1 abundance by WB analysis in a new cohort of BALF samples and respective serum samples, reporting an interesting result. In IPF BALF, we visualize the up-regulation of a band at 25 kDa, corresponding to the differential protein species that we found by proteomics, while in serum samples the same band shows an opposite trend (Figure 8A). Moreover, WB highlights a characteristic band at a higher molecular weight (50 kDa), which was found to be highly abundant in both BALF and serum of IPF patients. In order to exclude the presence of APOA1 proteoforms at 50 kDa as aspecific signals and, therefore, to evaluate their real presence, we performed a 2DWB of a IPF BALF. As expected, several protein species were evidenced by the WB of IPF BALF, not only at 25 kDa but also at 50 kDa (Figure 8B).

**Figure 9:**
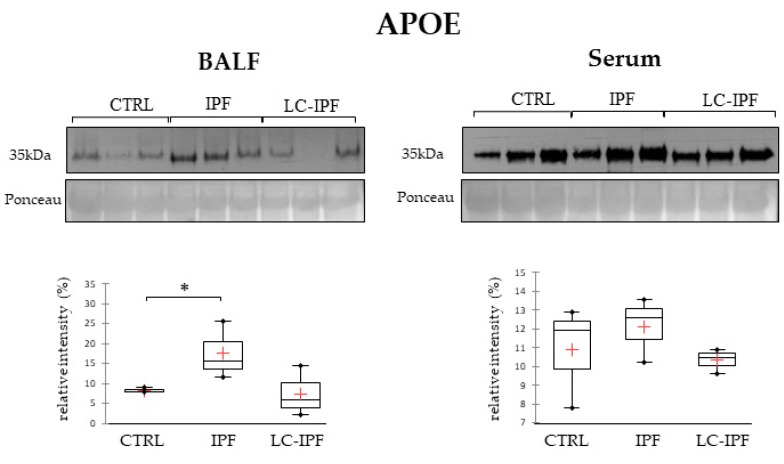
Western blot analysis of APOE in BALF and serum samples. Statistical analysis was performed by Kruskal–Wallis test and Dunn correction and reported in the box plots for CTRL–IPF–LC-IPF (^∗^ *p*-value < 0.05). Ponceau red is reported to assure the equal loaded amount of sample and for the intensity normalization. BALF and serum samples used for WB were three for CTRL, three for IPF, and three for LC-IPF.

Due to the interesting finding regarding APOA1 and our previous evidence regarding the contribution of dysregulated lipid metabolism in fibrotic processes and in IPF development [20,26], we also decided to test apolipoprotein E (APOE) in BALF and serum samples, in light of apolipoproteins involvement with dependent pathways aimed at modulating normal lung health and the pathogenesis of respiratory diseases, including asthma, acute lung injury, cancer, emphysema, pulmonary fibrosis, and pulmonary hypertension [27]. As reported in Figure 9, APOE levels significantly increase in IPF BALF samples, with respect to CTRLs, while there are no modifications at the serum level.

### 2.5. Pearson’s Correlation of Differential Proteins Highlighted a Relationship between Inflammation and Lipid Metabolism and Transport in IPF and LC-IPF and the Crossroad Role of NAMPT 

Furthermore, we performed a Pearson’s correlation analysis, aiming to investigate whether the identified proteins present a linear relationship about their regulation. We constructed a correlation matrix containing pairwise relationships between all identified proteins (Figure S2) and the significant correlations are highlighted in bold (*p* < 0.05). Correlation analysis reveals a co-regulation of proteins involved in organization of the actin cytoskeleton, cell motility, and adhesion. In particular, PROF1 was positively correlated with S10A6 and GDIR1, corroborating our bioinformatic results. Moreover, proteins involved in inflammation, such as S10A9, S10A6, B2MG and CYTB, were positively correlated with each other and to proteins engaged in lipid metabolism and transport, for example to FAB4; on the other hand, CFAB and FETUA were co-regulated with APOA1. TTHY and RET4 were interrelated and negatively correlated to immune and inflammatory response proteins, such as FRIL, IGHA1, and IGKC. 

**Figure 10:**
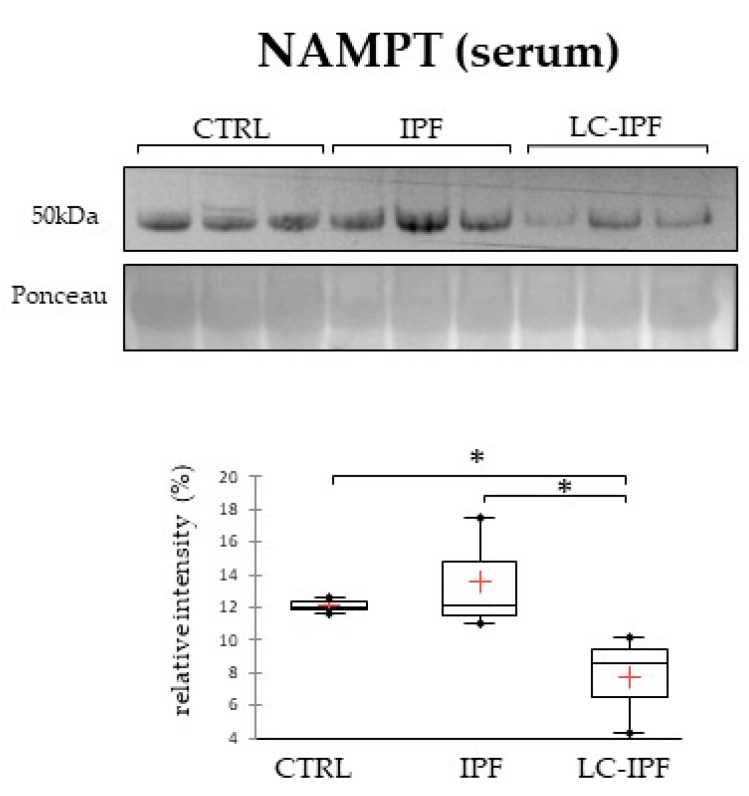
Western blot analysis of NAMPT in serum samples. Statistical analysis was performed by Kruskal–Wallis test and Dunn correction and reported in the box plots for CTRL–IPF–LC-IPF (^∗^ *p*-value ≤ 0.05). Ponceau red is reported to assure the equal loaded amount of sample. Samples used were three for CTRL, three for IPF, and three for LC-IPF.

Enrichment analysis by process networks combined with correlation analysis reveals that the up-regulated proteins in both IPF and LC-IPF are particularly involved in inflammatory pathways and also in lipid metabolism. Given these results, we propose to evaluate the nicotinamide phosphoribosyltransferase (NAMPT) in BALF and serum samples. In fact, the extracellular NAMPT is reported to exhibit cytokine-/adipokine like properties, suggesting that it stands at the crossroad between lipid metabolism and inflammation [28]. Figure 10 reports 1DWB results performed only in serum samples because NAMPT was not detectable in BALF samples. In serum, NAMPT is lower abundant in LC-IPF with respect to IPF, distinguishing the two conditions. 

**Figure 11:**
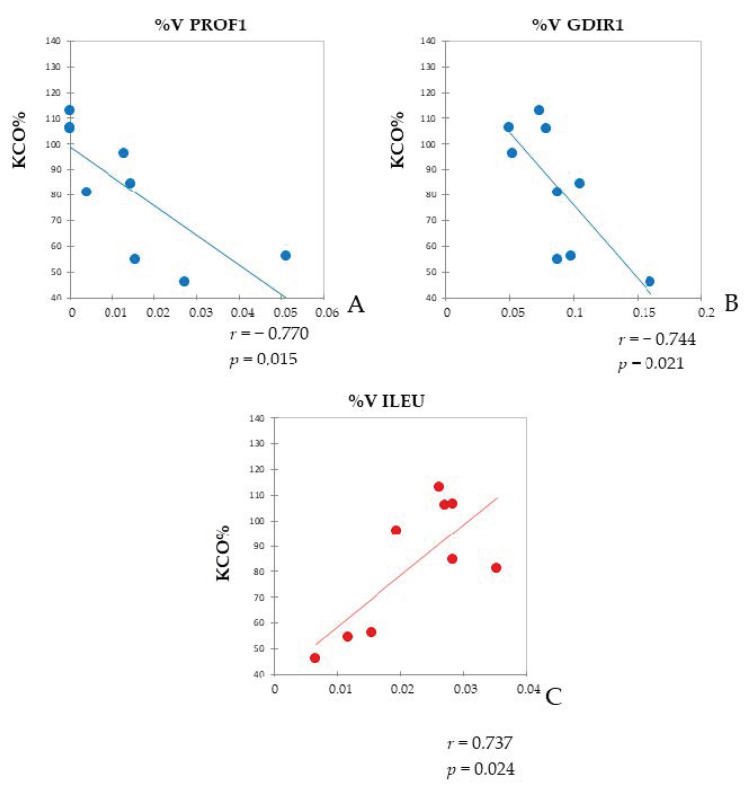
Pearson’s Correlation. Pearson’s correlation between KCO% in IPF and LC-IPF patients and PROF1 %V (**A**), GDIR1 %V (**B**), and ILEU %V (**C**) in BALF samples. Pearson’s correlation coefficients (r) and *p*-values (p) are shown for each plot.

Moreover, we evaluated whether KCO% (approximately kCO/barometric pressure in mL/min/mmHg/L) is correlated with the %V of significant proteins detected by proteomics. PROF1 and Rho GDP-dissociation inhibitor 1 (GDIR1) were negatively correlated with KCO% (Pearson’s correlation coefficients r = −0.744; r = −0.770, respectively) (*p* < 0.05) (Figure 11A,B). On the other hand, the %V of leukocyte elastase inhibitor (ILEU) was positively correlated with KCO% (Pearson’s correlation coefficient r = 0.737) (*p* < 0.05) (Figure 11C).

## 3. Discussion

IPF is a pathology associated with poor prognosis and patients that are also diagnosed with LC have a further reduced mean survival time (1.6–1.7 years) [7,12,13]. For this reason, the necessity to identify early diagnostic biomarkers in addition to clinical characteristics of lung cancer in IPF is essential in order to establish screening protocols and diagnostic and therapeutic strategies. Moreover, histological or cytological diagnosis of lung cancer is an invasive procedure, which is sometimes impossible due to the severe respiratory condition of the patients, considering also that a radiological diagnosis alone is often not enough. For these reasons, retrospective analyses of BALF of IPF patients and IPF that developed LC could be helpful to identify differential biomarkers. Moreover, a further evaluation at the serum level might permit one to measure these potential biomarkers with a less invasive procedure more suitable for such compromised patients. 

Proteomic analysis, performed on BALF samples of patients with diagnosed IPF and LC-IPF, together with healthy controls, represents a good strategy to identify a pool of proteins that, changing in abundance, could characterize the presence of LC in IPF lung. By the unsupervised multivariate analysis, BALF proteome comparison in these three conditions (CTRL, IPF, LC-IPF) immediately shows that the protein pattern from all IPF patients, including LC, is distinctive from healthy controls. On the other hand, both the supervised PCA and heatmap show a distinction between IPF and LC-IPF protein profiles suggesting that the presence of a tumor induces a change in protein abundance, distinctive from IPF pathology alone. Enrichment analysis reveals several terms related to HDL-mediated reverse cholesterol and sphingolipid transport and lipoprotein metabolism, response to hypoxia and oxidative stress, and inflammation, associated with both IPF and LC-IPF. These results confirmed the pivotal role of the lipid metabolism and inflammation in the pathogenesis of IPF, as already found by Landi et al. in 2014 and 2020 [2,26], while LC-IPF up-regulated proteins are mainly related to inflammation by IL-6 signaling, cell adhesion and regulation of cytoskeleton rearrangement, retinoic acid regulation, and its role in the initiation of transcription.

In order to explore connections between the identified proteins in a complementary way to functional interrelation, we generated a correlation matrix by Pearson’s coefficient. By proteomic analysis, TTHY and RET4 were strongly up-regulated in LC-IPF condition. They were not only interrelated, but also presented the same behaviour with respect to FRIL, HPT, IGHA1, and IGKC, suggesting their strong co-regulation. In addition to the interrelation found between TTHY and RET4, we interestingly highlight a canonical path starting from TTHY to E2F by protein network analysis. All these results could suggest the pivotal role of TTHY in LC-IPF. Indeed, TTHY is well-known to form a complex with RET4 that, in turn, binds retinol required for RET4-mediated transport from the liver to target tissues [29,30]. RET4-TTHY complex dysregulation is associated with metabolic diseases [31,32,33] and with cancer risks [34,35,36,37]. Moreover, TTHY is able to bind thyroid hormones [38,39] for their transport in different districts and binding with their receptors. Thyroid hormone receptor alpha (TR-alpha), suggested by our network, interacts with the E2F1 promoter regulating its transcription to control G1- to S-phase transition [40].

Furthermore, our correlation and bioinformatic analyses highlight that several proteins involved in inflammation present a direct correlation with proteins associated with lipid metabolism and transport, strengthening the coexisting functional link between metabolic dysregulation and inflammation, which occur in IPF and LC-IPF. Additionally, we conducted a correlation test between dysregulated proteins and KCO% as clinical functional parameter, because it better reflects the pulmonary physiology [41]. ILEU is positively correlated with KCO%; indeed, it is reported to protect the cell from proteases released during stress conditions. According to our proteomic analysis, ILEU is up-regulated in both IPF and LC-IPF compared to CTRL. Of note, LC-IPF presents decreased levels of ILEU with respect to IPF, although not significantly. Dysregulated levels of ILEU are associated with the migratory and invasive properties of cancer cells [42,43,44,45,46]. 

On the other hand, two proteins were negatively correlated with KCO%, such as PROF1 and GDIR1, which were co-regulated and associated with cell adhesion [47], as suggested by enrichment analysis. PROF1 is an actin binding protein involved in cellular homeostasis and cytoskeletal structure maintenance. It influences actin polymerization modulating cell migration metastases, and increases cell survival [48,49,50,51]. In accordance with our results, Almatroodi et al. report higher levels of PROF1 in BALF samples of patients with primary lung adenocarcinoma [52] and Allen et al. demonstrates that higher levels of intracellular PROF1 are accompanied by an increased level of extracellular PROF1, suggesting its ability to serve as an extracellular agent, irrespective of the cell type of origin [53]. According to these data, we also identified PROF1 in extracellular vesicles purified by IPF BALF [54]. Therefore, the extracellular PROF1 in the tumour microenvironment may serve as a paracrine mediator, manipulating cells biological behaviour and affecting tumorigenesis. 

Consistent with our proteomic analysis, an increased level of GDIR1 has been found in multiple types of human cancers, including lung cancer tissues [55,56,57]. Hence, GDIR1 and PROF1 could represent two biomarkers of LC development in IPF patients and may become an attractive target for anticancer treatments, since they are related to cell migration and adhesion. 

Another interesting differential protein is calgranulin B (S10A9), a central hub of our interactomes and already found up-regulated in IPF in our previous works [58,59]. Interestingly, this protein, highly abundant in IPF, further increases in the presence of lung adenocarcinoma. Our MetaCore network reports PROF1 is linked to HIF-1α [60], which in turn regulates the transcription of S10A9. Indeed, hypoxic stimuli characteristic of IPF trigger a variety of adaptive responses [26], which promote many key mechanisms of tumorigenesis and cancer progression and contribute to the aggressive tumor behavior [61,62]. The involvement of S10A9 in IPF pathogenesis has been extensively discussed in previous works as a biomarker of poor prognosis [58,63,64]. According to our bioinformatic analyses performed by MetaCore, S10A9 is involved in inflammation processes in pulmonary cancer and may play a key role in inflammation-associated cancer [65,66]. For this reason, S10A9 might be a biomarker of LC onset following the presence of IPF, since it further increases in BALF and serum samples from LC-IPF patients [64,65,67,68]. S10A9 is often co-expressed with S100A8 in lung cancer, forming a heterodimer called calprotectin [67,69]. Besides that, evidence shows that a high abundance of calprotectin could be one of the main characteristics of lung cancer cells with bone metastatic potential, because it might increase the release of IL6 by binding with Toll-like receptor 4 on bone marrow adipocytes, as suggested by our MetaCore network. In addition, the activation of TLR4 could induce IL6 production in macrophages [69] and mesenchymal stem cells [70]. Additionally, NF-kB or STAT3 participate in the release of IL-6 induced by S10A9 [71].

Looking at the network resulting from this comparison, HNF-4α interestingly emerges as a pivotal transcriptional factor. In particular, it is a nuclear receptor whose ligands are fatty acids and generally HNF-4α target genes encoded proteins are involved in metabolic processes [14]. In our network, FABP physically and functionally interacts with HNF-4α, which in turn directly regulates the majority of the other proteins up-regulated in LC-IPF, such as TTHY, ALBU, RET4, and FETUA; the last two are also known as hepatokines linked to the induction of metabolic dysfunctions [48]. Indeed, HNF-4α is also reported to exert an oncogenic role by the induction of the Warburg effect [72]. Moreover, HNF-4α is reported as part of the signature genes of invasive lung mucinous adenocarcinoma, together with FOXA3, SPDEF and mucins, such as MUC5AC, MUC5B, and MUC3. For all these reasons, HNF-4α is worthy of further studies since it might be considered a prognostic biological marker for IPF-associated lung adenocarcinoma and might be implicated in IPF molecular mechanisms. 

While the pathways related to cellular metabolism and adhesion and retinoic acid regulation are statistically relevant in LC-IPF, suggesting dysregulation of cellular differentiation, the lipid metabolism and transport acquire a similar significance in both LC-IPF and IPF. Therefore, it is interesting to verify whether lipid dysregulation could be a biomarker for IPF with respect to LC-IPF by testing APOA1 and APOE. Both proteins were found to be highly abundant in BALF IPF and lower in LC-IPF. Interestingly, the behaviour of APOA1 was opposite in serum samples of the same IPF BALF donors. While APOA1 could be a characteristic biomarker in both BALF and serum samples, APOE does not change its abundance at the serum level. Of note, we report an unexpected result for APOA1 by 1DWB showing differential protein species at a higher MW (50 kDa) in IPF BALF and serum, subsequently also evidenced by 2DEWB. 

On one hand, the proteins suggesting a dysregulated lipid metabolism profile are characteristic in distinguishing IPF to LC-IPF and, on the other hand, up-regulated proteins in LC-IPF are particularly linked to inflammatory pathways. Given this, we propose the evaluation of NAMPT level on BALF and serum samples in IPF, LC-IPF, and CTRLs, considering that extracellular NAMPT is reported to exhibit cytokine-/adipokine like-properties, suggesting that it stands at the crossroad between metabolism and inflammation [28]. Our results do not report NAMPT in BALF samples; rather, it is detected only in serum, evidencing a lower abundance in LC-IPF with respect to IPF and CTRLs. NAMPT is frequently studied in lung cancer, particularly in NSCLC, and it is considered a potential target of treatment [73,74], as well as a prognostic biomarker [75]. Its characteristic low abundance in serum samples of LC-IPF patients could be tested in a wider cohort of samples in order to render our data more robust.

The main limitation of the present study is the limited number of samples; therefore an expansion of these studies to a larger cohort could confirm our findings and further demonstrate the utility of TTHY, RET4, PROF1, S10A9 and GDIR1, and NAMPT as biomarkers of LC onset in IPF patients.

## 4. Materials and Methods

### 4.1. Population, Bronchoscopy, and Samples Collection 

The study group included 23 patients diagnosed with IPF (17 males, mean age 75 ± 6.1 years, 6 ex-smokers and 11 no-smoker; 6 females of mean age 76 ± 15, 1 ex-smoker). They were picked from patients who were naïve at the moment of the diagnosis. None of them ever received previous treatment with anti-fibrotic drugs. Seven patients diagnosed with both IPF and lung cancer (6 males of mean age 63 ± 9 years, 3 ex-smokers and 3 no-smoker; 1 female of age 80, no-smoker), without any prior exposure to chemotherapy or radiotherapy, were selected for the study. IPF diagnosis was performed according to the ATS/ERS guidelines at Siena Regional Referral Centre for Interstitial Lung Diseases. BAL test was performed for diagnostic purposes, according to multidisciplinary evaluation. The histocytopatological assessment of LC was perfomed through cytological analysis of malignant pleural effusion, transbronchial biopsy, and CT-guided needle aspiration (2, 1 and 5 patients, respectively). Demographic data and information such as smoking habits, onset symptoms and comorbidities were recorded in a database, as well as BAL differential cell count, blood gas analysis data, and lung function tests. In addition, functional, radiological, histological, and immunological data were also recorded. According to ATS/ERS guidelines, pulmonary functionality tests were performed in order to obtain FEV1, FVC, TLC, and DLCO percentages [6], and high-resolution computed tomography of the chest (HRCT) was performed, then interpretation was conducted by experienced radiologists. Diagnosis was performed in a context of multidisciplinary discussion. Fifteen healthy adults were included in the analysis, BAL samples from healthy workers exposed to asbestos were used as controls, and the inclusion criteria were: normal values of lung function parameters, not suffering chronic or infection diseases, and not on treatment for any illness. To be included in the study, no asbestos fibres or bodies could be reported through BAL examination and no diagnosis of asbestosis and/or pleural plaques could be be reported at the end of multidisciplinary discussion. Pack-years or other measures of cigarette smoking were not part of inclusion criteria. All patients transmitted written informed consent to be included in the study, which was approved by the local ethics committee (OSS_REOS code number 12908; C.E.A.V.S.E. Markerlung 17431, 15 June 2020). After collection of informed consents, bronchoalveolar lavage was performed in order to exclude other interstitial lung diseases. 

Part of the cohort was used for proteomic analysis by random selection and the resting samples were used for validations by WB. 

BAL samples were collected through standardized protocols, introducing aliquots of normal saline solution by fibrobronchoscope (Olympus IT-10; Olympus Italia, Milan, Italy). The first aliquot re-collected did not undergo immunological analysis, while the. The others were centrifuged for 5 min at 800× *g*, allowing the cells to separate from the fluid component. Cells differential count was performed on cytocentrifuge preparations, in particular BAL lymphocytes’ phenotype was analysed by flow cytometry (FACSCalibur; Becton and Dickinson, Milan, Italy) and monoclonal antibodies (anti-CD3, CD4, CD8, CD69; Becton and Dickinson). Fluidic part of BAL (BALF) was stored at –80 °C and used for proteomics.

In the morning blood samples were collected, into serum tubes (BD vacutainer, SST II Advance, Plymouth UK) after 8 h of fasting, then they were centrifuged for 10 min at 1690× *g*. Fluidic part of BAL (BALF) and serum samples were stored and stored at −80 °C. 

### 4.2. BALF Preparation for Two-Dimensional Electrophoresis

BALF samples were dialyzed for 12 h at 4 °C against four changes of distilled water, in order to clean samples from salts. As a second step, they were first lyophilized and then dissolved in lysis buffer, composed of 8 M urea, 4% *w*/*v* 3-[(3-cholamidopropyl) dimethylammonia]-1-propanesulfonate hydrate (CHAPS), 40 mM Tris base, and 1% *w*/*v* dithioerythritol (DTE). Protein concentration was determined by Bradford assay [76], 60 µg of protein was loaded for the analytical run, and 600 µg for the MS-preparative run, with traces of bromophenol blue.

### 4.3. Two-Dimensional Electrophoresis

Two-dimensional electrophoresis (2DE) was performed by the Immobiline polyacrylamide system. The first dimension was carried out on pH 3–10 nonlinear, 18 cm long, immobilized pH gradient strips (Cytiva, formerly GE Healthcare) using an Ettan™ IPGphor™ system (GE Healthcare, Uppsala, Sweden). Analytical runs consisted of an overnight rehydration of strips with 350 µL of lysis buffer and traces of bromophenol blue at room temperature and a loading of the samples at the cathodic end of the strips by cup-loading. Electrical conditions were as follows: 200 V for 8 h, from 200 to 3500 V in 2 h, 3500 V for 2 h, from 3500 to 5000 V in 2 h, 5000 V for 3 h, from 5000 to 8000 V in 1 h, 8000 V for 3 h, from 8000 to 10,000 V in 1 h, 10,000 V, for a total of 90,000 VhT (total Volts per hour) at 16 °C. Preparative runs were carried out by rehydration loading with 350 µL of the sample at 16 °C at 30 V overnight, then 100 µL of the sample were loaded at the cathodic end of the strips, with the following electrical conditions: 200 V for 8 h, from 200 to 3500 V in 2 h, 3500 V for 2 h, from 3500 to 5000 V in 2 h, 5000 V for 3 h, from 5000 to 8000 V in 1 h, 8000 V for 3 h, from 8000 to 10,000 V in 1 h, 10,000 V, for a total of 90,000 VhT (total Volts per hour) at 16 °C. Traces of bromophenol blue and carrier ampholyte at 0.2% for the analytical runs and at 2% for the preparative ones were added to the samples. After isoelectric focusing, an equilibration of the strips was performed as follows: first, incubation for 12 min in a solution composed of 6 M urea, 2% *w*/*v* SDS, 2% *w*/*v* DTE, 30% *v*/*v* glycerol and 0.5 M Tris–HCl pH 6.8 and a second incubation for 5 min—in a solution composed of 6 M urea, 2% *w*/*v* SDS, 2.5% *w*/*v* iodoacetamide, 30% *v*/*v* glycerol, 0.5 M Tris–HCl pH 6.8 and traces of bromophenol blue. Second dimension by SDS/PAGE was performed at 9 °C at 40 mA/gel constant current, using 9–16% SDS polyacrylamide linear gradient gels (18 cm × 20 cm × 1.5 mm in size). Then, ammoniacal silver staining was used for analytical gels, whereas MS-compatible silver staining was used for preparative gels. The resulting gels were scanned with Image Scanner III laser densitometer run by the LabScan 6.0 software (GE Healthcare). Image Master 2D Platinum 6.0 software (GE Healthcare, Uppsala, Sweden) was used to carry out 2D image analysis. 

### 4.4. Statistical Analysis

Proteomic data were statistically analyzed by XLSTAT 2021.1.1 (by Addinsoft, Paris, France) applying non-parametrical tests (Kruskal–Wallis and Dunn’s multiple tests). Moreover, the percentage of relative volume (%V) comparison of the 2DE protein spots among the three conditions was performed taking into consideration at least a 1.8-fold change in the ratio of the %V means and a *p*-value ≤ 0.05. Unsupervised and supervised Principal Component Analysis (PCA) and Heatmap analysis were performed by XLSTAT 2021.1.1. Venn diagram was performed by Venny 2.0 free online software (https://bioinfogp.cnb.csic.es/tools/venny/index2.0.2.html; accessed on 10 October 2021). A correlation test by Pearson’s correlation test was performed to investigate the linear correlations between %V of the proteins of interest, and to determine the linear relationship between the percentage of carbon monoxide transfer coefficient (KCO%, is approximately kCO/barometric pressure in mL/minute/mmHg/L) and the %V of the proteins of interest, by using software XLStat.

### 4.5. MALDI-TOF Identification by PMF

Differential electrophoretic spots were manually excised from MS-preparative gels. Spots were subjected to destaining by using a solution of 30 mM potassium ferricyanure and 100 mM sodium tiosulphate anhydrous and then in 200 mM ammonium bicarbonate. The destained spots were then dehydrated in 100% acetonitrile (ACN). Overnight rehydration in trypsin solution at 37 °C of spots was performed to digest proteins. The obtained peptide solution was spotted on the MALDI target (0.75 µL), dried, and covered with 1 µL of matrix solution, composed of 5 mg/mL α-cyano-4-hydroxycinnamic acid (CHCA) dissolved in 50% *v*/*v* ACN and 5% *v*/*v* trifluoroacetic acid (TFA), then dried again. UltrafleXtreme™ MALDI-ToF/ToF instrument (Bruker Corporation, Billerica, MA, USA) was used for MS analysis, according to Landi C et al. [20]. Spectra were acquired by FlexControl software and then analyzed with the Flex Analysis software version 3.0 (Bruker). Acquired spectra were calibrated using auto-proteolytic tripsin peptides as internal standards. Filtering of the resulting mass lists was performed to remove contaminant, such as mass matrix-related ions, keratin-derived peaks and trypsin auto-lysis peptides peaks. Peptide Mass Fingerprinting (PMF) search was performed using MASCOT (Matrix Science Ltd., London, UK, http://www.matrixscience.com, accessed on 22 September 2021), setting the search parameters as follows: Swiss-Prot/TrEMBL and NCBInr were chosen as databases, *Homo sapiens* as taxonomy, a peptide mass tolerance of 100 ppm, only one missed cleavage site, carbamidomethylation (iodoacetamide alkylation) of cysteine as fixed modification, and oxidation of methionine as a variable modification. The mass spectrometry proteomics data have been deposited to the ProteomeXchange Consortium via the PRIDE [77] partner repository with the dataset identifier PXD037720.

### 4.6. MetaCore Enrichment Analysis

Enrichment analysis was performed by submitting the list of the identified proteins by their accession numbers to the MetaCore version 22.1 build (http://portal.genego.com, Clarivate Analytics, Philadelphia, PA, USA, accessed on 5 January 2022). MetaCore works by a revised database of human protein–protein and protein–DNA interactions, transcription factors, signaling and metabolic pathways, disease and toxicity, and the effects of bioactive molecules. Specifically, we first performed enrichment analysis obtaining a hierarchical list of pathway maps and network processes, organized by their statistical significance (*p* ≤ 0.001). Furthermore, we performed a network analysis processing our proteins list by shortest path algorithm, which builds a hypothetical network of the potential interactions between the experimental proteins and the MetaCore database proteins.

### 4.7. Western Blot Analysis 

Mono-dimensional Western blot analysis was performed with a cohort of BALF and serum samples of CTRL, IPF, and LC-IPF patients. Tested proteins were apolipoprotein A1 (sc376811-Santa Cruz Biotechnology, Dallas, TX, USA), apolipoprotein E (sc53570- Santa Cruz Biotechnology), profilin 1 (P7624-Sigma-Aldrich, St. Louis, MO, USA), calgranulin B (S10A9) (SAB1409623-Sigma-Aldrich), and nicotinamide phosphoribosyltransferase (NAMPT)(cat. 61122-Cell Signaling and Technology, Danvers, MA, USA). Additionally, a two-dimensional Western blot for apolipoprotein A1 was performed. Aliquots of BALF samples were prepared as previously described. Serum samples were prepared as reported by Landi C et al. [78]. Considering mono-dimensional WB, samples were diluted in Laemmli buffer (100 mM Tris–HCl pH 6.8, 2% (*w*/*v*) SDS, 20% (*v*/*v*) glycerol, 4% (*v*/*v*) β-mercaptoethanol) and heated at 95 °C for five minutes. Specifically, twenty-five μg of proteins for each sample were loaded and separated on 10% polyacrylamide gel. In order to increase resolution and patients’ number, for calgranulin B validation a bigger 1D-WB (18 cm × 20 cm × 1.5 mm in size) was performed, 40 μg of proteins for each sample were loaded and separated on 10% polyacrylamide gel. As described by Towbin [79], gels were transferred onto a nitrocellulose membrane (Hybond ECL, GE Healthcare) [79]. Hybridization with primary antibodies was carried out overnight at room temperature. Goat peroxidase-conjugated anti-mouse or anti-rabbit immunoglobulin G (Sigma, working dilution 1:3000) secondary antibody was incubated for 2 h at room temperature. Furthermore, 2DE-WB was carried out, resolving 120 μg of sample using one patient per condition (IPF, LC-IPF, CTRL). Western blot preparative gels were transferred onto nitrocellulose overnight at a total current of 2 Å, at 4 °C. One- and two-dimensional membranes were reversibly stained with Ponceau red, composed as 0.2% *w*/*v* Ponceau S in 3% *w*/*v* trichloroacetic acid, in order to confirm correct protein transfer. Hybridization with primary antibodies was carried out overnight at room temperature. Goat-anti-mouse or anti-rabbit HRP-conjugate secondary antibody was incubated for 2 h at room temperature. Immunostained bands and spots were visualized by chemoluminescence. Band and spot density were quantified by Image Master 2D Platinum and statistical analysis was performed by XL STAT tool.

## 5. Conclusions

The proteomic analysis of BALF from IPF, LC-IPF, and CTRL patients provided insights into the molecular pathways and processes differently regulated in the two pathologic conditions. Most of the enriched pathways common in the IPF and LC-IPF conditions were related to HDL-mediated reverse cholesterol and sphingolipid transport and lipoprotein metabolism, response to hypoxia and oxidative stress, and inflammation. Moreover, the correlation test stressed the relationship of inflammatory response and lipid metabolism and transport in pathologic conditions, further confirmed by the APOA1, APOE, and NAMPT dysregulation. In addition, the down-regulation of the latter in LC-IPF could be considered as potential prognostic biomarker. This study also emphasizes the different proteomic patterns that occur in LC-IPF compared to IPF and CTRL. In particular, the marked up-regulation of RET4 associated with TTHY may represent a potential biomarker of LC development in IPF disease, principally related to metabolic dysfunction and to the activation of pathways dependent on retinoic acid receptors. The correlation analysis between proteomic data and functional parameters provided a functional and clinical context for data interpretation. The KCO% was negatively correlated with PROF1 and GDRI, and we confirmed the up-regulation of PROF1 in LC-IPF BALF, which could represent an interesting biomarker of LC associated with IPF.

## Figures and Tables

**Table 1:** Demographic features, radiological data, respiratory functional parameters, and BAL cellular analysis among IPF, LC-IPF and healthy subjects. FVC: forced vital capacity; FEV1: forced expiratory volume in the first second; DLCO: diffusion lung capacity for carbon monoxide; KCO: approximately kCO/barometric pressure in mL/minute/mmHg/L; HRCT: high resolution computed tomography; PFT: pulmonary function test.

Parameters	IPF	LC-IPF	Healthy Controls	*p*-Value
**N°**	23	7	15	
**Age (yrs)**	75 ± 6.1	63 ± 9	54.3 ± 12.9	0.0010
**Male gender (%)**	17	6	5	0.177
**Smoking status**				
**- Current**	0	0	1	0.854
**- Former**	12	3	4	
**- Never**	11	4	10	
**HRCT pattern**				
**- UIP**	20	6	n.a	0.954
**- Probable UIP**	3	1	n.a	
**PFTs**				
**- FVC (mL)**	2482 ± 804.2	2505 ± 688	n.a	0.8611
**- FVC (% p.v.)**	75.6 ± 18.6	63.5 ± 15.8	n.a	0.1994
**- FEV1 (mL)**	1952 ± 712.1	1920 ± 420.9	n.a	0.8013
**- FEV1 (% p.v.)**	78 ± 21.4	64.3 ± 17.4	n.a	0.179
**- DLCO (% p.v.)**	49.1 ± 21.4	36.3 ± 13.5	n.a	0.2772
**- KCO (% p.v.)**	89.4 ± 24.8	68.8 ± 23.5	n.a	0.272
**BAL cellular analysis**				
**- % Macrophage**	74.5 ± 21.1	65 ± 21.9	82 ± 15.3	0.0257
**- % Lymphocytes**	12.2 ± 9.5	12 ± 15.5	15.6 ± 12.1	0.2598
**- % Neutrophils**	11.2 ± 12.6	18 ± 17.1	5.4 ± 6.3	0.0140
**- % Eosinophils**	2.7 ± 4.1	5.6 ± 6.2	2.2 ± 1.6	0.1568

**Table 2:** Identification results by PMF of the statistically relevant spots, detected by proteomic analysis on BALF samples. Spot number, corresponding to that in Figure 1 and Figure 2, UniProt protein name, entry name and accession number, MetaCore name, pI and MW and Mascot search results such as score, matched peptides, and coverage % were reported.

						Mascot Search Results		
Spot n°	Protein Name	Entry Name	Accession Number	MetaCore Name	pI MW	Score	Matched Peptides	Coverage (%)
**3**	Immunoglobulin heavy constant alpha 1 Fragment N	IGHA1_HUMAN	P01876	IGHA1, IgA, IgA1	6.08–38,486	184	14/18	28
**5**	Ferritin light chain	FRIL_HUMAN	P02792	FTL	5.51–20,064	187	18/47	68
**7**	Hemoglobin subunit alpha	HBA_HUMAN	P69905	Alpha1-globin	8.72–15,305	181	10/12	69
**10**	Retinol-binding protein 4	RET4_HUMAN	P02753	RBP4	5.76–23,337	136	8/14	52
**13**	Immunoglobulin heavy constant alpha 1 Fragment N	IGHA1_HUMAN	P01876	IGHA1, IgA, IgA1	6.08–38,486	163	11/13	25
**14**	Haptoglobin	HPT_HUMAN	P00738	HP, HP-alpha, HP-beta	6.13–45,861	123	9/17	20
**15**	Hemoglobin subunit beta	HBB_HUMAN	P68871	VV-Hemorphin-7	6.75–16,102	138	8/10	61
**16**	Hemoglobin subunit alpha	HBA_HUMAN	P69905	Alpha1-globin	8.72–15,305	135	7/7	61
**17**	Beta-2-microglobulin	B2MG_HUMAN	P61769	Beta-2-microglobulin	6.06–13,820	250	11/11	87
**19**	Fatty acid-binding protein, adipocyte	FABP4_HUMAN	P15090	Fatty acid-binding protein	6.59–14,824	207	13/18	68
**22**	Beta-2-microglobulin	B2MG_HUMAN	P61769	Beta-2-microglobulin	6.06–13,820	84	4/5	44
**25**	Annexin A3	ANXA3_HUMAN	P12429	Annexin III	5.63–36,524	86	6/6	17
**28**	Hemoglobin subunit alpha Fragment N	HBA_HUMAN	P69905	Alpha1-globin	8.72–15,305	81	5/6	34
**29**	Protein S100-A9	S10A9_HUMAN	P06702	Calgranulin B	5.71–13,291	151	7/9	84
**30**	Protein S100-A6	S10A6_HUMAN	P06703	Calcyclin	5.33–10,230	111	10/13	40
**31**	Albumin fragment C	ALBU_HUMAN	P02768	Albumin	5.92–71,317	135	11/18	18
**32**	Immunoglobulin kappa constant	IGKC_HUMAN	P01834	IGKC	6.11–11,929	132	6/7	59
**33**	Rho GDP-dissociation inhibitor 1	GDIR1_HUMAN	P52565	RhoGDI alpha	5.01–23,207	109	7/12	31
**34**	Apolipoprotein A-I	APOA1_HUMAN	P02647	HDL, Apoa1	5.56–30,759	268	20/28	53
**35**	Immunoglobulin kappa constant	IGKC_HUMAN	P01834	IGKC	6.11–11,929	101	5/8	53
**36**	Alpha-2-HS-glycoprotein	FETUA_HUMAN	P02765	Fetuin-A, AHSG-A, AHSG-B	5.43–40,114	168	16/32	40
**37**	Transthyretin	TTHY_HUMAN	P02766	Transthyretin	5.52–15,991	130	7/13	72
**38**	Transthyretin	TTHY_HUMAN	P02766	Transthyretin	5.52–15,991	131	7/13	57
**40**	Transthyretin	TTHY_HUMAN	P02766	Transthyretin	5.52–15,991	147	7/9	64
**42**	Apolipoprotein A-I	APOA1_HUMAN	P02647	HDL, Apoa1	5.56–30,759	174	13/21	37
**43**	Protein S100-A11	S10AB_HUMAN	P31949	S100	6.56–11,847	138	8/9	58
**46**	Haptoglobin	HPT_HUMAN	P00738	HP, HP-alpha, HP-beta	6.13–45,861	149	9/11	24
**53**	Peroxiredoxin-1	PRDX1_HUMAN	Q06830	Peroxiredoxin	8.25–223,225	286	16/18	62
**55**	Immunoglobulin heavy constant gamma	IGHG1_HUMAN	P01857	IGHG1, IgG, IgG1	8.46–36,596	130	8/11	37
**57**	Albumin Fragment C	ALBU_HUMAN	P02768	Albumin	5.92–71,317	129	11/13	19
**60**	Immunoglobulin kappa constant	IGKC_HUMAN	P01834	IGKC	6.11–11,929	76	4/9	48
**61**	Immunoglobulin kappa constant	IGKC_HUMAN	P01835	IGKC	6.11–11,929	137	7/12	78
**63**	Profilin-1	PROF1_HUMAN	P07737	Profilin	8.44–15,216	261	17/19	75
**65**	Cystatin	CYTB_HUMAN	P04080	Cystatin B	6.96–11,190	93	5/10	58
**67**	Haptoglobin	HPT_HUMAN	P00738	HP, HP-alpha, HP-beta	6.13–45,861	232	18/23	34
**68**	Apolipoprotein A-I	APOA1_HUMAN	P02647	HDL, Apoa1	5.56–30,759	248	19/28	49
**69**	MIX: Pulmonary surfactant-associated protein A2	SFPA2_HUMAN	Q8IWL1	SP-A2	5.07–26,622	146	10/22	45
	Pulmonary surfactant-associated protein A1	SFTA1_HUMAN	Q8IWL2	SP-A	5.07–26,623	146	10/22	45
	Transthyretin	TTHY_HUMAN	P02766	Transthyretin	5.52–15,991	90	6/22	71
**72**	Complement factor B	CFAB_HUMAN	P00751	Factor B, Factor Ba, Factor Bb	6.67–86,847	273	21/25	30
**73**	Alpha-2-HS-glycoprotein	FETUA_HUMAN	P02765	Fetuin-A, AHSG-A, AHSG-B	5.43–40,114	94	7/13	19
**74**	Calcyphosin	CAYP1_HUMAN	Q13938	CAPS, Calcyphosin	6.90–23,650	346	22/26	53
**76**	Immunoglobulin kappa constant	IGKC_HUMAN	P01834		6.11–11,929	98	5/9	55
**77**	Leukocyte elastase inhibitor	ILEU_HUMAN	P30740	SERPINB1	5.90–42,829	76	5/7	15
**78**	Serotransferrin	TRFE_HUMAN	P02787	Holotransferrin	6.81–79,294	506	42/53	49
**82**	Immunoglobulin kappa constant	IGKC_HUMAN	P01834	IGKC	6.11–11,929	118	6/10	59
**86**	Apolipoprotein A-I	APOA1_HUMAN	P02647	HDL, Apoa1	5.56–30,759	175	12/16	28
**87**	Immunoglobulin heavy constant gamma	IGHG1_HUMAN	P01857	IGHG1, IgG, IgG1	8.46–36,596	94	6/9	27

## Data Availability

The mass spectrometry proteomics data have been deposited to the ProteomeXchange Consortium via the PRIDE partner repository with the dataset identifier PXD037720.

## Supplementary Materials

The following supporting information can be downloaded at: https://www.mdpi.com/article/10.3390/ijms24020925/s1.

## Abbreviations

**%V**	percentage relative volume
**1DEWB**	1-Dimensional Western Blot
**2-DE**	Two-Dimensional Electrophoresis
**2DEWB**	Two-Dimensional Western Blot
**AACT**	Alpha-1-antichymotrypsin
**ACN**	acetonitrile
**ADC**	Adenocarcinoma
**ALBU**	Albumin
**ANXA3**	Annexin A3
**APOA1**	Apolipoprotein A1
**APOE**	Apolipoprotein E
**ATS/ERS**	American Thoracic Society/European Respiratory Society
**B2MG**	Beta-2-Microglobulin
**BAL**	Bronchoalveolar Lavage
**BALF**	Fluidic part of BAL
**BCR**	B Cell Antigen Receptor
**BMP**	Bone Morphogenetic Protein
**C/EBP**	CCAAT-Enhancer-Binding Proteins
**CAYP1**	Calcyphosin
**CFAB**	Complement Factor B
**CHAPS**	3-((3-cholamidopropyl) dimethylammonio)-1-propanesulfonate
**CHCA**	α-cyano-4-hydroxycinnamic acid
**CTRL**	Healthy Controls
**CYTB**	Cystatin
**DASs**	Differentially Abundant Spots
**DLCO**	Diffusing Capacity Of The Lungs For Carbon Monoxide
**DTE**	1,4-Dithioerythritol
**E2F1**	E2F Transcription Factor 1
**ESR1**	Estrogen Receptor 1
**FABP4**	Fatty Acid Binding Protein 4
**FEV1**	Forced Expiratory Volume in the 1st second
**FETUA**	Alpha-2-HS-glycoprotein
**FOXA3**	Forkhead Box A3
**FRIL**	Ferritin Light Chain
**FVC**	Forced Vital Capacity
**GDIR1**	rho-specific guanine nucleotide dissociation inhibitor α
**HRCT**	High-resolution computed tomography of the chest
**HIF-1A**	Hypoxia-Inducible Factor 1-Alpha
**HBA**	Hemoglobin subunit alpha
**HBB**	Hemoglobin subunit beta
**HDL**	High Density Lipoproteins
**HNF-4α**	Hepatocyte Nuclear Factor 4 Alpha
**HP**	Haptoglobin
**HRCT**	High-resolution computed tomography
**IGHA1**	Immunoglobulin Heavy Constant Alpha 1
**IGHG1**	Immunoglobulin Heavy Constant Gamma
**IGKC**	Immunoglobulin Kappa Constant
**IL-6**	Interleukin-6
**ILDs**	Interstitial Lung Diseases
**ILEU**	Leukocyte Elastase Inhibitor
**IPF**	Idiopathic Pulmonary Fibrosis
**LC**	Lung Cancer
**LC-IPF**	Lung Cancer associated with Idiopathic Pulmonary Fibrosis
**MALDI**	Matrix Assisted Laser Desorption/Ionization
**MW**	Molecular Weight
**NAMPT**	Nicotinamide Phosphoribosyltransferase
**NF-Kb**	Nuclear Factor Kappa-Light-Chain-Enhancer of Activated B Cells
**NSCLC**	Non-Small Cell Lung Cancer
**PC1**	First Principal Component
**PC2**	Second Principal Component
**PC3**	Third Principal Component
**PCA**	Principal Component Analysis
**PCs**	Principal Components
**PFT**	Pulmonary Function Test
**pI**	Isoelectric Point
**PMF**	Peptide Mass Fingerprinting
**PRDX1**	Peroxiredoxin 1
**PROF1**	Profilin 1
**RBP4**	Binding Protein 4
**RET4**	Retinol Binding Protein 4
**S10A9**	Calgranulin B
**S10AB**	Protein S100-A11
**SFTA**	Pulmonary Surfactant-Associated Protein A
**SDS**	Sodium Dodecyl Sulfate
**SP-A**	Surfactant A2
**SPDEF**	SAM Pointed Domain-Containing Ets Transcription Factor
**STAT3**	Signal Transducer and Activator Of Transcription 3
**TLC**	Total Lung Capacity
**TFA**	Trifluoroacetic Acid
**TLR4**	Toll-like receptor 4
**ToF**	Time Of Flight
**TR-alpha**	Thyroid Hormone Receptor Alpha
**TRFE**	Serotransferrin
**TTHY**	Transthyretin
**WB**	Western Blot
