# Supplementary material for: BAL Proteomic Signature of Lung Adenocarcinoma in IPF Patients and Its Transposition in Serum Samples for Less Invasive Diagnostic Procedures
Source: Int J Mol Sci. 2023 Jan 4;24(2):925. doi: 10.3390/ijms24020925 (PMC9861565; doi:10.3390/ijms24020925)
Supplement: Supplementary file 1 [file ijms-24-00925-s001.zip › Table S1.pdf]

				Kruskal-Wallis	mean			Ratios					
**Spot n°**	**Protein name**	**Entry name**	**AC**	**p-value**	**IPF**	**LC-IPF**	**CTRL**	**IPF/ LC-IPF**	**LC-IPF/ IPF**	**IPF/CTRL**	**CTRL/IPF**	**LC-IPF/ CTRL**	**CTRL/ LC-IPF**
**3**	Immunoglobulin heavy constant alpha 1 Fragment N	IGHA1_HUMAN	P01876	0.005	0.036	0.000	0.000	**0.036**	**0**	**0.036**	**0**	**0**	**0**
**4**				0.005	0.008	0.000	0.000	**0.008**	**0**	**0.008**	**0**	**0**	**0**
**5**	Ferritin light chain	FRIL_HUMAN	P02792	0.005	0.045	0.000	0.000	**0.045**	**0**	**0.045**	**0**	**0**	**0**
**6**				0.005	0.016	0.000	0.000	**0.016**	**0**	**0.016**	**0**	**0**	**0**
**7**	Hemoglobin subunit alpha	HBA_HUMAN	P69905	0.005	0.045	0.000	0.000	**0.045**	**0**	**0.045**	**0**	**0**	**0**
**8**				0.005	0.029	0.000	0.000	**0.029**	**0**	**0.029**	**0**	**0**	**0**
**10**	Retinol-binding protein 4	RET4_HUMAN	P02753	0.006	0.061	0.158	0.024	0.403	**2.480**	**2.712**	0.369	**6.726**	0.149
**11**				0.006	0.021	0.064	0.000	0.320	**3.121**	**0.021**	**0**	**0.064**	**0**
**12**				0.006	0.043	0.022	0.000	**2.317**	0.432	**0.043**	**0**	**0.022**	**0**
**13**	Immunoglobulin heavy constant alpha 1 Fragment N	IGHA1_HUMAN	P01876	0.006	0.027	0.000	0.000	**0.027**	**0**	**0.027**	**0**	**0**	**0**
**14**	Haptoglobin	HPT_HUMAN	P00738	0.006	0.037	0.000	0.000	**0.037**	**0**	**0.037**	**0**	**0**	**0**
**15**	Hemoglobin subunit beta	HBB_HUMAN	P68871	0.006	0.126	0.374	0.000	0.397	**2.521**	**0.126**	**0**	**0.374**	**0**
**16**	Hemoglobin subunit alpha	HBA_HUMAN	P69905	0.006	0.052	0.019	0.000	**2.994**	0.334	**0.052**	**0**	**0.019**	**0**
**17**	Beta-2-microglobulin	B2MG_HUMAN	P61769	0.006	0.043	0.059	0.000	0.734	1.363	**0.043**	**0**	**0.059**	**0**
**18**				0.006	0.020	0.006	0.000	**3.390**	0.295	**0.020**	**0**	**0.006**	**0**
**19**	Fatty acid-binding protein. adipocyte	FABP4_HUMAN	P15090	0.006	0.107	0.062	0.000	**2.071**	0.483	**0.107**	**0**	**0.062**	**0**
**22**	Beta-2-microglobulin	B2MG_HUMAN	P61769	0.006	0.046	0.039	0.000	1.158	0.864	**0.046**	**0**	**0.039**	**0**
**23**				0.006	0.055	0.095	0.000	0.662	1.510	**0.055**	**0**	**0.095**	**0**
**24**				0.006	0.010	0.008	0.000	1.141	0.876	**0.010**	**0**	**0.008**	**0**
**25**	Annexin A3	ANXA3_HUMAN	P12429	0.006	0.031	0.041	0.003	0.831	1.203	**12.745**	0.078	**15.335**	0.065
**26**				0.006	0.029	0.023	0.000	1.457	0.686	**0.029**	**0**	**0.023**	**0**
**27**				0.006	0.041	0.043	0.000	1.119	0.893	**0.041**	**0**	**0.043**	**0**
**28**	Hemoglobin subunit alpha Fragment N	HBA_HUMAN	P69905	0.006	0.077	0.069	0.000	1.110	0.901	**0.077**	**0**	**0.069**	**0**
**29**	Protein S100-A9	S10A9_HUMAN	P06702	0.006	0.058	0.073	0.000	0.928	1.077	**0.058**	**0**	**0.073**	**0**
**30**	Protein S100-A6	S10A6_HUMAN	P06703	0.006	0.096	0.105	0.000	0.915	1.092	**0.096**	**0**	**0.105**	**0**
**31**	Albumin fragment C	ALBU_HUMAN	P02768	0.007	0.271	0.464	0.008	0.636	1.572	**38.409**	0.026	**60.397**	0.017
**32**	Immunoglobulin kappa constant	IGKC_HUMAN	P01834	0.007	1.063	0.892	0.224	1.163	0.860	**4.634**	0.216	**3.985**	0.251
**33**	Rho GDP-dissociation inhibitor 1	GDIR1_HUMAN	P52565	0.007	0.074	0.076	0.016	0.778	1.285	**3.659**	0.273	**4.701**	0.213
**34**	Apolipoprotein A-I	APOA1_HUMAN	P02647	0.007	0.228	0.243	0.013	0.914	1.094	**17.465**	0.057	**19.114**	0.052

**35**	Immunoglobulin kappa constant	IGKC_HUMAN	P01834	0.007	0.795	0.392	0.073	**1.868**	0.535	**9.977**	0.100	**5.342**	0.187
**36**	Alpha-2-HS-glycoprotein	FETUA_HUMAN	P02765	0.007	0.041	0.053	0.000	0.865	1.155	**0.041**	**0**	**0.053**	**0**
**37**	Transthyretin	TTHY_HUMAN	P02766	0.007	0.066	0.124	0.025	0.519	**1.928**	**2.572**	0.389	**4.960**	0.202
**38**	Transthyretin	TTHY_HUMAN	P02766	0.007	0.104	0.271	0.045	0.334	**2.992**	**1.989**	0.503	**5.951**	0.168
**40**	Transthyretin	TTHY_HUMAN	P02766	0.007	0.029	0.000	0.000	**0.029**	**0**	**0.029**	**0**	**0**	**0**
**41**				0.007	0.010	0.127	0.000	0.081	**12.385**	**0.010**	**0**	0.127	**0**
**42**	Apolipoprotein A-I	APOA1_HUMAN	P02647	0.007	0.083	0.112	0.007	0.752	1.330	**11.572**	0.086	**15.388**	0.065
**43**	Protein S100-A11	S10AB_HUMAN	P31949	0.007	0.018	0.014	0.000	1.180	0.848	**0.018**	**0**	**0.014**	**0**
**46**	Haptoglobin	HPT_HUMAN	P00738	0.007	0.060	0.049	0.000	1.018	0.983	**0.060**	**0**	**0.049**	**0**
**53**	Peroxiredoxin-1	PRDX1_HUMAN	Q06830	0.012	0.032	0.037	0.000	0.797	1.255	**0.032**	**0**	**0.037**	**0**
**55**	Immunoglobulin heavy constant gamma	IGHG1_HUMAN	P01857	0.012	0.088	0.196	0.013	0.486	**2.057**	**7.222**	0.138	**14.854**	0.067
**57**	Albumin Fragment C	ALBU_HUMAN	P02768	0.012	0.022	0.130	0.072	0.166	**6.027**	0.301	**3.325**	**1.813**	0.552
**60**	Immunoglobulin kappa constant	IGKC_HUMAN	P01834	0.012	0.405	0.167	0.040	**2.230**	0.448	**9.418**	0.106	**4.223**	0.237
**61**	Immunoglobulin kappa constant	IGKC_HUMAN	P01835	0.012	0.948	0.830	0.446	1.179	0.848	**2.194**	0.456	**1.860**	0.538
**62**				0.012	0.012	0.026	0.000	0.507	**1.971**	**0.012**	**0**	**0.026**	**0**
**63**	Profilin-1	PROF1_HUMAN	P07737	0.013	0.007	0.022	0.000	0.139	**7.188**	**0.007**	**0**	**0.022**	**0**
**65**	Cystatin	CYTB_HUMAN	P04080	0.015	0.136	0.152	0.022	0.959	1.043	**6.672**	0.150	**6.957**	0.144
**66**				0.022	0.074	0.049	0.015	1.666	0.600	**5.299**	0.189	**3.181**	0.314
**67**	Haptoglobin	HPT_HUMAN	P00738	0.023	0.344	0.621	0.200	0.564	1.772	1.753	0.571	**3.106**	0.322
**68**	Apolipoprotein A-I	APOA1_HUMAN	P02647	0.025	0.119	0.145	0.016	0.874	1.144	**7.982**	0.125	**9.134**	0.109
	MIX: Pulmonary surfactant-associated protein A2	SFPA2_HUMAN	Q8IWL1										
**69**	Pulmonary surfactant-associated protein A1	SFTA1_HUMAN	Q8IWL2	0.025	0.821	0.222	0.254	**3.915**	0.255	**3.426**	0.292	0.875	1.143
	Transthyretin	TTHY_HUMAN	P02766										
**70**				0.027	0.041	0.060	0.012	0.764	1.310	**3.682**	0.272	**4.822**	0.207
**72**	Complement factor B	CFAB_HUMAN	P00751	0.032	0.050	0.052	0.013	0.865	1.157	**3.509**	0.285	**4.059**	0.246
**73**	Alpha-2-HS-glycoprotein	FETUA_HUMAN	P02765	0.035	0.040	0.072	0.013	0.553	**1.810**	**3.049**	0.328	**5.518**	0.181
**74**	Calcyphosin	CAYP1_HUMAN	Q13938	0.036	0.118	0.047	0.030	**2.476**	0.404	**3.828**	0.261	1.546	0.647
**76**	Immunoglobulin kappa constant	IGKC_HUMAN	P01834	0.038	1.163	0.524	0.780	**2.271**	0.440	1.525	0.656	0.671	1.489
**77**	Leukocyte elastase inhibitor	ILEU_HUMAN	P30740	0.038	0.025	0.018	0.008	1.509	0.663	**3.467**	0.288	**2.298**	0.435
**78**	Serotransferrin	TRFE_HUMAN	P02787	0.040	0.562	0.656	0.939	0.771	1.297	0.539	**1.856**	0.699	1.431
**82**	Immunoglobulin kappa constant	IGKC_HUMAN	P01834	0.043	0.709	0.269	0.237	**2.917**	0.343	**3.314**	0.302	1.136	0.880
**84**				0.045	0.021	0.011	0.006	**2.089**	0.479	**3.922**	0.255	**1.877**	0.533

**86**	Apolipoprotein A-I	APOA1_HUMAN	P02647	0.046	0.137	0.103	0.045	1.372	0.729	**3.152**	0.317	**2.298**	0.435
**87**	Immunoglobulin heavy constant gamma	IGHG1_HUMAN	P01857	0.047	0.087	0.077	0.022	1.159	0.863	**4.106**	0.244	**3.542**	0.282

Table S1. Table reporting the statistically relevant spots by spot numbers, protein name, UniProt accession number, Kruskal Wallis p-values, IPF, LC-IPF, CTRL means and ratios. Grey squares highlight qualitative differences and the values refer to the means of %V of the condition in which the relative spot was present.
