# Supplementary material for: BAL Proteomic Signature of Lung Adenocarcinoma in IPF Patients and Its Transposition in Serum Samples for Less Invasive Diagnostic Procedures
Source: Int J Mol Sci. 2023 Jan 4;24(2):925. doi: 10.3390/ijms24020925 (PMC9861565; doi:10.3390/ijms24020925)
Supplement: Supplementary file 1 [file ijms-24-00925-s001.zip › Table S2 Process Networks.pdf]

PROCESS NETWORK

#	Networks	Total	min(pValue)	Min FDR	LC-IPF				IPF			
					p-value	FDR	In Data	Network Objects from Active Data	p-value	FDR	In Data	Network Objects from Active Data
1	Inflammation_IL-6 signaling	119	1,98E-05	0,000534	1,98E-05	0,000534	5	HP/HB complex, HDL proteins, HP, Alpha1-globin, HBB	0,000293	0,007039	4	HP/HB complex, HDL proteins, HP, Alpha1-globin
2	Immune response_BCR pathway	137	0,007211	0,097351	0,007211	0,097351	3	Profilin, Kappa chain (Ig light chain), IGHG1	0,055808	0,299789	2	Kappa chain (Ig light chain), IGHG1
3	Inflammation_Complement system	75	0,018436	0,119946	0,020239	0,119946	2	Factor Bb, Factor B	0,018436	0,221233	2	Factor Bb, Factor B
4	Cell adhesion_Integrin-mediated cell-matrix adhesion	214	0,023946	0,119946	0,023946	0,119946	3	RhoGDI alpha, Profilin, Profilin I	0,457617	0,492118	1	RhoGDI alpha
5	Immune response_Phagocytosis	223	0,026655	0,119946	0,026655	0,119946	3	Profilin, Profilin I, HDL proteins	0,471613	0,492118	1	HDL proteins
6	Immune response_Phagosome in antigen presentation	241	0,032552	0,125559	0,032552	0,125559	3	Beta-2-microglobulin, Profilin, Profilin I	0,498583	0,498583	1	Beta-2-microglobulin
7	Development_Hemopoiesis, Erythropoietin pathway	136	0,060139	0,200377	0,060139	0,200377	2	Alpha1-globin, HBB	0,320653	0,491672	1	Alpha1-globin
8	Development_Ossification and bone remodeling	157	0,070964	0,200377	0,077331	0,200377	2	PRDX1, Fetuin-A	0,070964	0,299789	2	PRDX1, Fetuin-A
9	Response to hypoxia and oxidative stress	161	0,074145	0,200377	0,080768	0,200377	2	Peroxiredoxin, PRDX1	0,074145	0,299789	2	Peroxiredoxin, PRDX1
10	Cell adhesion_Glycoconjugates	162	0,074947	0,200377	0,081635	0,200377	2	RhoGDI alpha, HP	0,074947	0,299789	2	RhoGDI alpha, HP
11	Inflammation_Histamine signaling	215	0,121133	0,253284	0,131333	0,253284	2	Kappa chain (Ig light chain), IGHG1	0,121133	0,403006	2	Kappa chain (Ig light chain), IGHG1
12	Development_Neurogenesis_Axonal guidance	229	0,134335	0,261847	0,145471	0,261847	2	RhoGDI alpha, Calcyclin	0,134335	0,403006	2	RhoGDI alpha, Calcyclin
13	Immune response_Th17-derived cytokines	98	0,242583	0,406073	0,253048	0,406073	1	Calgranulin B	0,242583	0,491672	1	Calgranulin B
14	Inflammation_IFN-gamma signaling	109	0,266018	0,406073	0,277297	0,406073	1	Factor B	0,266018	0,491672	1	Factor B
15	Inflammation_IL-4 signaling	115	0,278508	0,406073	0,290206	0,406073	1	IGHG1	0,278508	0,491672	1	IGHG1
16	Inflammation_Amphoterin signaling	118	0,284677	0,406073	0,296577	0,406073	1	Calgranulin B	0,284677	0,491672	1	Calgranulin B
17	Inflammation_Inflammasome	120	0,288762	0,406073	0,300795	0,406073	1	APOA1	0,288762	0,491672	1	APOA1
18	Inflammation_MIF signaling	141	0,330337	0,441834	0,343649	0,441834	1	PRDX1	0,330337	0,491672	1	PRDX1
19	Proliferation_Negative regulation of cell proliferation	183	0,406663	0,446038	0,421971	0,446038	1	Calgizzarin	0,406663	0,491672	1	Calgizzarin
20	Transport_Calcium transport	192	0,421906	0,446038	0,437554	0,446038	1	Annexin III	0,421906	0,491672	1	Annexin III
21	Immune response_Antigen presentation	193	0,423577	0,446038	0,439261	0,446038	1	Beta-2-microglobulin	0,423577	0,491672	1	Beta-2-microglobulin
22	Cell adhesion_Amyloid proteins	197	0,430213	0,446038	0,446038	0,446038	1	RhoGDI alpha	0,430213	0,491672	1	RhoGDI alpha

Table S2. MetaCore Analysis: the comparison of Process Network by gene ontology of the up-regulated protein in LC-IPF (black) and up-regulated proteins in IPF (grey).
