# Supplementary material for: BAL Proteomic Signature of Lung Adenocarcinoma in IPF Patients and Its Transposition in Serum Samples for Less Invasive Diagnostic Procedures
Source: Int J Mol Sci. 2023 Jan 4;24(2):925. doi: 10.3390/ijms24020925 (PMC9861565; doi:10.3390/ijms24020925)
Supplement: Supplementary file 1 [file ijms-24-00925-s001.zip › Table S3 Pathways Maps.pdf]

Enrichment by Pathway Maps												
#	Maps	Total	min(pValue)	Min FDR	LC-IPF				IPF			
					p-value	FDR	In Data	Network Objects from Active Data	p-value	FDR	In Data	Network Objects from Active Data
1	HDL dyslipidemia in type 2 diabetes and metabolic syndrome X	38	6,21E-07	4,35E-05	8,83E-07	5,59E-05	4	HDL, Pre beta-1 HDL, APOA1, Nascent HDL	6,21E-07	4,35E-05	4	HDL, Pre beta-1 HDL, APOA1, Nascent HDL
2	Transport_HDL-mediated reverse cholesterol transport	42	9,36E-07	4,35E-05	1,33E-06	5,59E-05	4	HDL, Pre beta-1 HDL, APOA1, Nascent HDL	9,36E-07	4,35E-05	4	HDL, Pre beta-1 HDL, APOA1, Nascent HDL
3	Alternative complement cascade disruption in age-related macular degeneration	31	2,38E-05	0,000737	3,08E-05	0,000861	3	Factor Ba, Factor Bb, Factor B	2,38E-05	0,000737	3	Factor Ba, Factor Bb, Factor B
4	Immune response_Alternative complement pathway	53	0,000121	0,002804	0,000156	0,003269	3	Factor Ba, Factor Bb, Factor B	0,000121	0,002804	3	Factor Ba, Factor Bb, Factor B
5	Lipoprotein metabolism	68	0,000253	0,004713	0,000327	0,005485	3	Pre beta-1 HDL, APOA1, Nascent HDL	0,000253	0,004713	3	Pre beta-1 HDL, APOA1, Nascent HDL
6	Retinoic acid regulation of oligodendrocyte differentiation in multiple sclerosis	24	0,001035	0,013481	0,001035	0,013481	2	RBP4, Transthyretin	0,043342	0,137987	1	Transthyretin
7	Fenofibrate in treatment of type 2 diabetes and metabolic syndrome X	25	0,001123	0,013481	0,001123	0,013481	2	RBP4, APOA1	0,045108	0,137987	1	APOA1
8	Cholesterol and Sphingolipid transport / Recycling to plasma membrane in lung (normal and CF)	29	0,00128	0,015886	0,001513	0,015886	2	HDL, APOA1	0,00128	0,019833	2	HDL, APOA1
9	Development_FGF and BMP signaling in early embryonic hepatogenesis	31	0,001729	0,016133	0,001729	0,016133	2	Transthyretin, Albumin	0,055641	0,137987	1	Transthyretin
10	Transcription_Role of the non-genomic action of Retinoic acid and phosphorylation of Retinoic acid receptors in the initiation of transcription	35	0,002201	0,018136	0,002201	0,018136	2	RBP4, Transthyretin	0,0626	0,137987	1	Transthyretin
11	Development_Retinoic acid and retinoic acid receptors in regulation of oligodendrocyte differentiation	37	0,002458	0,018136	0,002458	0,018136	2	RBP4, Transthyretin	0,066062	0,137987	1	Transthyretin
12	Transcription_Role of AP-1 in regulation of cellular metabolism	38	0,002591	0,018136	0,002591	0,018136	2	Alpha1-globin, HBB	0,067788	0,137987	1	Alpha1-globin
13	Niacin-HDL metabolism	46	0,0032	0,022664	0,003777	0,022664	2	HDL proteins, APOA1	0,0032	0,042521	2	HDL proteins, APOA1
14	Development_EPO-induced PI3K/AKT pathway and Ca(2+) influx	43	0,003308	0,021373	0,003308	0,021373	2	Alpha1-globin, HBB	0,076373	0,137987	1	Alpha1-globin

15	Development_Signaling pathways in embryonic hepatocyte maturation	51	0,004625	0,025899	0,004625	0,025899	2	RBP4, APOA1	0,089953	0,137987	1	APOA1
16	Transport_Intracellular cholesterol transport	83	0,010097	0,058654	0,01187	0,058654	2	HDL, APOA1	0,010097	0,117377	2	HDL, APOA1
17	Immune response _Sialic-acid receptors (Siglecs) signaling	14	0,025506	0,129242	0,027695	0,129242	1	HP	0,025506	0,137987	1	HP
18	Transition of Smoldering multiple myeloma to active multiple myeloma (schema)	17	0,03089	0,136222	0,033533	0,136222	1	Ig light chain	0,03089	0,137987	1	Ig light chain
19	Putative pathways of MHC class I-dependent postsynaptic long-term depression in major depressive disorder	21	0,038024	0,136222	0,041264	0,136222	1	Beta-2-microglobulin	0,038024	0,137987	1	Beta-2-microglobulin
20	Role of ZNF202 in regulation of expression of genes involved in atherosclerosis	21	0,038024	0,136222	0,041264	0,136222	1	HDL proteins	0,038024	0,137987	1	HDL proteins
21	Multiple sclerosis (general schema)	21	0,038024	0,136222	0,041264	0,136222	1	Transthyretin	0,038024	0,137987	1	Transthyretin
22	Transition of Monoclonal gammopathy of undetermined significance to active multiple myeloma (schema)	22	0,0398	0,136222	0,043188	0,136222	1	Ig light chain	0,0398	0,137987	1	Ig light chain
23	G-protein signaling_CDC42 inhibition	24	0,043342	0,136222	0,047024	0,136222	1	RhoGDI alpha	0,043342	0,137987	1	RhoGDI alpha
24	Putative pathways of activation of monoclonal protein secretion in multiple myeloma	25	0,045108	0,136222	0,048937	0,136222	1	Kappa chain (Ig light chain)	0,045108	0,137987	1	Kappa chain (Ig light chain)
25	Immune escape mechanisms in Prostate Cancer	25	0,045108	0,136222	0,048937	0,136222	1	Beta-2-microglobulin	0,045108	0,137987	1	Beta-2-microglobulin
26	Immune response_IFN-gamma actions on blood cells	28	0,050388	0,136222	0,054652	0,136222	1	Alpha1-globin	0,050388	0,137987	1	Alpha1-globin
27	Development_Role of CNTF and LIF in regulation of oligodendrocyte development	28	0,050388	0,136222	0,054652	0,136222	1	RhoGDI alpha	0,050388	0,137987	1	RhoGDI alpha
28	Cigarette smoke-mediated regulation of NRF2-antioxidant pathway in airway epithelial cells	29	0,052142	0,136222	0,05655	0,136222	1	PRDX1	0,052142	0,137987	1	PRDX1
29	Role of CNTF and LIF in regulation of oligodendrocyte development in multiple sclerosis	30	0,053893	0,136222	0,058444	0,136222	1	RhoGDI alpha	0,053893	0,137987	1	RhoGDI alpha
30	Regulation of lipid metabolism_RXR-dependent regulation of lipid metabolism via PPAR, RAR and VDR	30	0,053893	0,136222	0,058444	0,136222	1	APOA1	0,053893	0,137987	1	APOA1

31	G-protein signaling_Rac1 inhibition	31	0,055641	0,136222	0,060335	0,136222	1	RhoGDI alpha	0,055641	0,137987	1	RhoGDI alpha
32	Role of IL-6 in obesity and type 2 diabetes in adipocytes	32	0,057385	0,136222	0,062222	0,136222	1	HP	0,057385	0,137987	1	HP
33	Microsatellite instability in colorectal cancer	34	0,060865	0,136222	0,065984	0,136222	1	Beta-2-microglobulin	0,060865	0,137987	1	Beta-2-microglobulin
34	Role of growth factor receptors transactivation by Hyaluronic acid / CD44 signaling in tumor progression	35	0,0626	0,136222	0,06786	0,136222	1	RhoGDI alpha	0,0626	0,137987	1	RhoGDI alpha
35	Beta-catenin-dependent transcription regulation in colorectal cancer	36	0,064333	0,136222	0,069733	0,136222	1	Calcyclin	0,064333	0,137987	1	Calcyclin
36	Immune response_IL-6-induced acute-phase response in hepatocytes	36	0,064333	0,136222	0,069733	0,136222	1	HP	0,064333	0,137987	1	HP
37	G-protein signaling_Rac2 regulation pathway	36	0,064333	0,136222	0,069733	0,136222	1	RhoGDI alpha	0,064333	0,137987	1	RhoGDI alpha
38	Influence of low doses of Arsenite on Glucose stimulated Insulin secretion in pancreatic cells	36	0,064333	0,136222	0,069733	0,136222	1	PRDX1	0,064333	0,137987	1	PRDX1
39	iNKT cell-keratinocyte interactions in allergic contact dermatitis	36	0,064333	0,136222	0,069733	0,136222	1	Beta-2-microglobulin	0,064333	0,137987	1	Beta-2-microglobulin
40	Atorvastatin in treatment of type 2 diabetes and metabolic syndrome X	39	0,069511	0,137987	0,075328	0,142535	1	APOA1	0,069511	0,137987	1	APOA1
41	Development_TGF-beta family mediated differentiation of embryonic stem cells	42	0,074662	0,137987	0,080891	0,142535	1	Transthyretin	0,074662	0,137987	1	Transthyretin
42	Immune response_Antimicrobial actions of IFN-gamma	43	0,076373	0,137987	0,082739	0,142535	1	Factor B	0,076373	0,137987	1	Factor B
43	Cytoskeleton remodeling_Hyaluronic acid/ CD44 signaling pathways	43	0,076373	0,137987	0,082739	0,142535	1	RhoGDI alpha	0,076373	0,137987	1	RhoGDI alpha
44	Role of TNF-alpha, IL-1beta and IL-6 in development of obesity and type 2 diabetes in liver	45	0,079786	0,137987	0,086422	0,142535	1	APOA1	0,079786	0,137987	1	APOA1
45	Immune response_Induction of the antigen presentation machinery by IFN-gamma	53	0,093318	0,137987	0,101016	0,142535	1	Beta-2-microglobulin	0,093318	0,137987	1	Beta-2-microglobulin
46	Role of IL-23/ T17 pathogenic axis in psoriasis	53	0,093318	0,137987	0,101016	0,142535	1	Calgranulin B	0,093318	0,137987	1	Calgranulin B
47	Oxidative stress_Role of ASK1 under oxidative stress	54	0,094995	0,137987	0,102824	0,142535	1	PRDX1	0,094995	0,137987	1	PRDX1
48	Immune response_Antigen presentation by MHC class I, classical pathway	54	0,094995	0,137987	0,102824	0,142535	1	Beta-2-microglobulin	0,094995	0,137987	1	Beta-2-microglobulin

49	NRF2 regulation of oxidative stress response	54	0,094995	0,137987	0,102824	0,142535	1	PRDX1	0,094995	0,137987	1	PRDX1
50	Role of Thyroid hormone in regulation of oligodendrocyte differentiation in multiple sclerosis	55	0,09667	0,137987	0,104629	0,142535	1	Transthyretin	0,09667	0,137987	1	Transthyretin

Table S3. MetaCore Analysis: the comparison of Pathway Maps by gene ontology of the up-regulated protein in LC-IPF (black) and up-regulated proteins in IPF (grey).
