# Supplementary figures and images for: BAL Proteomic Signature of Lung Adenocarcinoma in IPF Patients and Its Transposition in Serum Samples for Less Invasive Diagnostic Procedures
Source: Int J Mol Sci. 2023 Jan 4;24(2):925. doi: 10.3390/ijms24020925 (PMC9861565; doi:10.3390/ijms24020925)

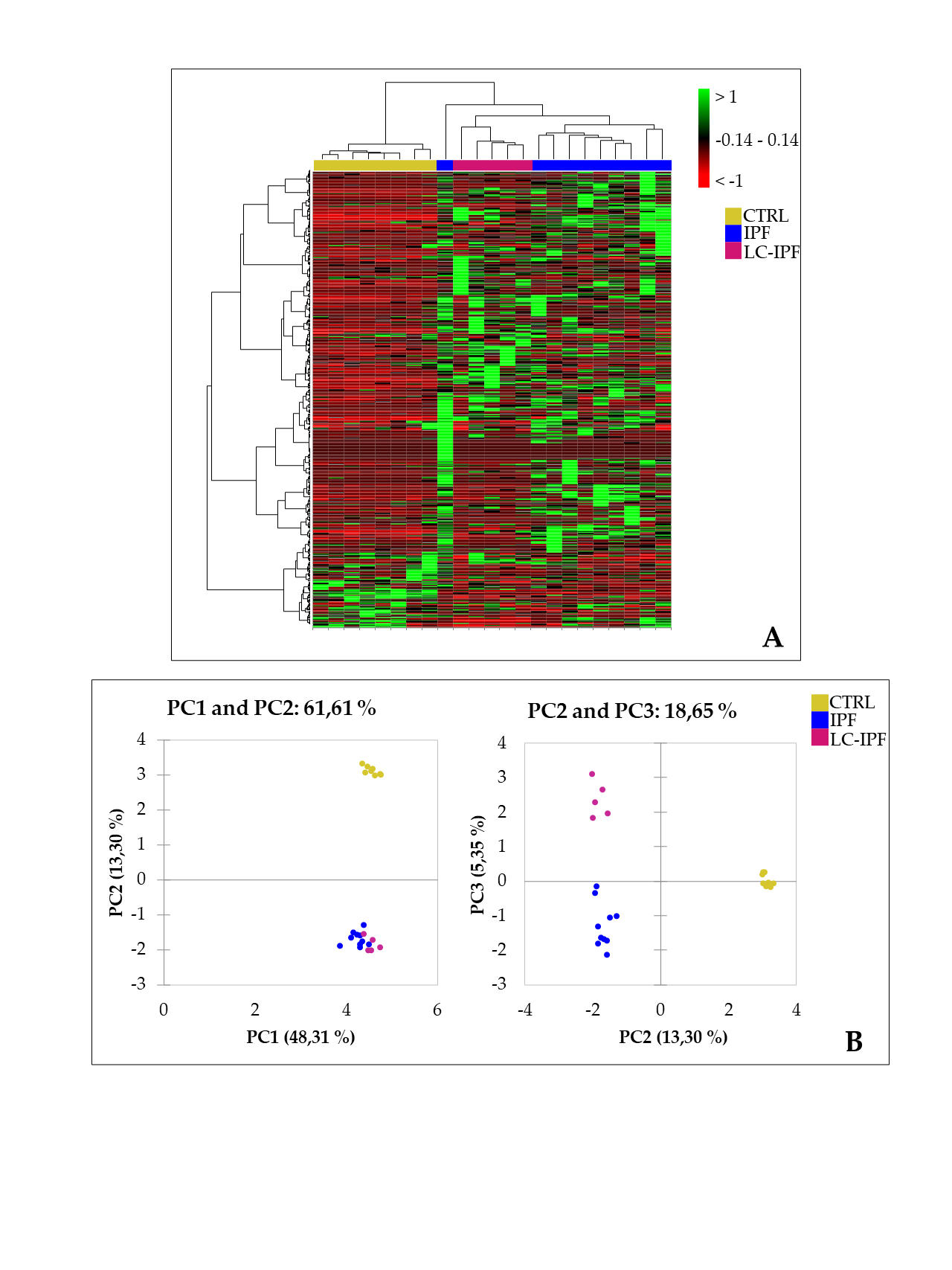

Supplement: Supplementary file 1 [file ijms-24-00925-s001.zip › Figure S1A and B.tif]

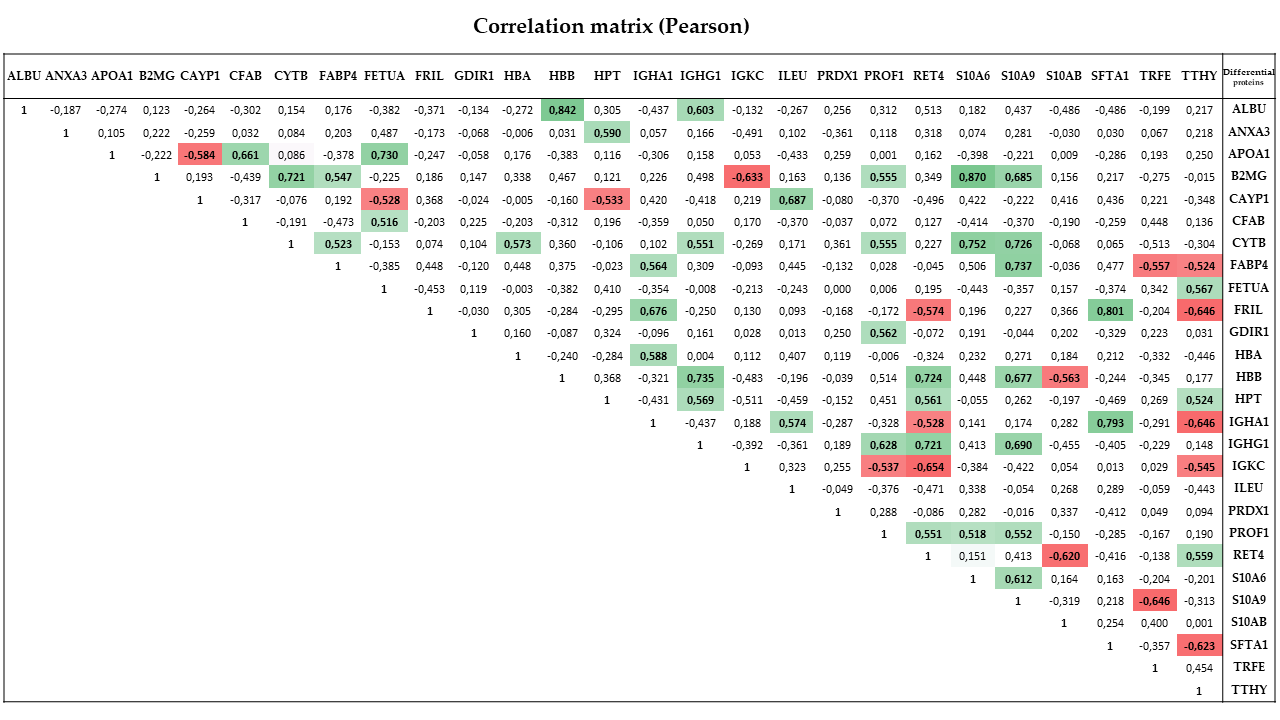

Supplement: Supplementary file 1 [file ijms-24-00925-s001.zip › Figure S2.tif]
